# Advances in *Eucommia ulmoides* polysaccharides: extraction, purification, structure, bioactivities and applications

**DOI:** 10.3389/fphar.2024.1421662

**Published:** 2024-08-16

**Authors:** Yanping Sun, Yuping Zhang, Minghao Sun, Wuyou Gao, Yujia He, Yu Wang, Bingyou Yang, Haixue Kuang

**Affiliations:** Key Laboratory of Basic and Application Research of Beiyao (Heilongjiang University of Chinese Medicine), Ministry of Education, Harbin, China

**Keywords:** Eucommia ulmoides polysaccharides, extraction and purification, structural characteristics, pharmacological activities, applications

## Abstract

*Eucommia ulmoides* (EU) is a precious tree species native to China originating during the ice age. This species has important economic value and comprehensive development potential, particularly in medicinal applications. The medicinal parts of EU are its bark (*Eucommiae cortex*) and leaves (*Eucommiae folium*) which have been successively used as a traditional Chinese medicine to treat diseases since the first century BC. During the last 2 decades, as natural polysaccharides have become of increasing interest in pharmacology, biomedicine, cosmetic and food applications, more and more scholars have begun to study polysaccharides derived from EU as well. EU polysaccharides have been found to have a variety of biological functions both *in vivo* and *in vitro*, including immunomodulatory, antioxidant, anti-inflammatory, anticomplementary, antifatigue, and hepatoprotective activities. This review aims to summarize these recent advances in extraction, purification, structural characteristics, pharmacological activities and applications in different fields of EU bark and leaf polysaccharides. It was found that both *Eucommiae folium* polysaccharides and *Eucommiae cortex* polysaccharides were suitable for medicinal use. *Eucommiae folium* may potentially be used to substitute for *Eucommiae cortex* in terms of immunomodulation and antioxidant activities. This study serves as a valuable reference for improving the comprehensive utilization of EU polysaccharides and further promoting the application of EU polysaccharides.

## 1 Introduction

*Eucommia ulmoides* (EU), also known as Formica, is a member of the monotypic family Eucommiaceae (Zhu et al., 2016). This species is a relic plant that survived the third glacial period and a dioecious perennial deciduous tree (2n = 34) grows up to approximately 20 m in height (Call and Dilcher, 1997; Wuyun et al., 2018; Jin et al., 2020; Liu et al., 2022). EU has the following typical botanical features: bark rough, beige or grayish-brown, with rubber inside, found to have strands of latex in bark openings; leaves simple ovate, 6–16 cm long and 3.5–6.5 cm wide, with serrate margins and acuminate apexes, found to have brown pilose when young, connected by elastic silvery white dense glue wires between the fracture surfaces and petioles; flower inconspicuous, small, greenish, male clustered, female axillary, with obovate bracts; flowering from April to May (He et al., 2014; Bao et al., 2024). EU is endemic and precious to China and is naturally distributed in southwest, southeast, central, and eastern southwest China to Zhejiang Province. The genuine producing areas of the EU are Sichuan, Shaanxi, Guizhou and Hubei provinces, and its habitats are protected by complex terrains (Dong et al., 2020; Xie et al., 2023). For a long time, EU has been a kind of tree with high economic value, which can be sold as Chinese medicinal materials and ornamental plants. At the same time, EU can also be used in the production of wood and its products. The “silver wire” found within the tree is a type of glial substance that exhibits adhesive properties and is resistant to corrosion by acids and alkalis. It is also characterized by its resistance to deformation, insulation, and heat insulation properties. In contemporary times, it can be refined into high-quality rubber or optical cable lines, submarine cables, oil pipelines, and other advanced materials. These attributes have earned it the moniker of “plant gold” (Zhao et al., 2024). In terms of medicine, EU has been used as a Chinese medicinal plant for over 2,000 years (Wang et al., 2011; Yao et al., 2012; Liu et al., 2016; Huang L. et al., 2021; Zhao et al., 2022), and it was first documented in Shen Nong Ben Cao Jing, a classical text on traditional Chinese medicine (Huang L. et al., 2021). Moreover, the Materia Medica Compendium systematically and comprehensively summarizes information related to EU (Xing et al., 2019). Currently, the Chinese Pharmacopoeia (2020 edition) includes EU bark (*Eucommiae cortex*) and EU leaves (*Eucommiae folium*) as two kinds of Chinese medicinal materials. Traditional Chinese medicine indicates that the properties and flavour of the *Eucommiae cortex* are sweet and warm, and the properties and flavour of *Eucommiae folium* are pungent and warm. Both materials are generally prepared as a general tonic to support liver and kidney health, promote muscle and bone strength, and alleviate hypertension. Importantly, the difference is that the *Eucommiae cortex* can also be used to calm the foetus (Liu et al., 2016; Huang L. et al., 2021). In recent years, the *Eucommiae cortex* has attracted much attention for its potential benefits in the treatment of diabetes mellitus, high blood pressure, hyperglycaemia, obesity, ageing, osteoporosis, Alzheimer’s disease, sexual dysfunction and other activities. These beneficial physiological effects are ascribed to a diverse array of bioactive ingredients, including cycloethers, phenols, flavonoids, lignans, polysaccharides, sterols, and plant gum (Luo et al., 2010; He et al., 2014; Xu et al., 2015; Hussain et al., 2016; Xing et al., 2019; Liu P. et al., 2020), and *Eucommiae folium* also contains these substances (Xing et al., 2019).

Polysaccharides are important macromolecules in the human body, made up of multiple monosaccharides linked together in complex structures (Amicucci et al., 2019; Tan et al., 2020). Numerous studies have identified natural polysaccharides from diverse biological sources, including animals, plants, and microorganisms, as confirmed by scientific research. These polysaccharides exhibit a variety of beneficial activities. In addition, they exhibit low toxicity and remarkable safety, making them an attractive therapeutic option for disease (Zhou et al., 2022). Studies of active ingredients in EU have found that polysaccharides from EU (EU polysaccharides) are considered some of the important effective components. With increasing interest in natural plant polysaccharides, research has been conducted on EU polysaccharides. Studies have demonstrated their antioxidant (Hong et al., 2013), immunoregulatory (Feng et al., 2016), anticomplementary (Zhu et al., 2008), anti-inflammatory (Sun et al., 2021), antifatigue (Xia and Piao, 2010), and hepatoprotective (Gao et al., 2020) activities, among others. For decades, various polysaccharides have been extracted from different EU parts (*Eucommiae cortex* and *Eucommiae folium*) by different extraction and purification methods. Because biological activity is related to chemical structure, it is important to analyze the chemical structure diversity of polysaccharides in EU to understand their biological activities (Tomoda, et al., 1990; Zhu et al., 2009; Feng H. et al., 2021).

However, although many scholars have studied EU polysaccharides, there is no systematic review of the polysaccharides of EU. As a result, there is a lack of clear understanding of the activity of polysaccharides from EU, and the structure-activity relationship is not well defined, which hinders the development of their applications. In this paper, we systematically summarized the current research results on EU polysaccharides and detailed the extraction, purification, structural analysis and pharmacological activity methods of EU polysaccharides. Then, the composition and biological properties of polysaccharides from the bark and leaves of EU were compared. In addition, bark harvesting requires the growth of the tree for several years, and improper harvesting methods readily cause tree death. This paper explores the potential for substituting polysaccharides from *Eucommiae cortex* with those from *Eucommiae folium* to conserve resources and further study the similar pharmacological effects of *Eucommiae* polysaccharides.

## 2 Search strategy

This review was systematically conducted thorough databases such as Google Scholar, Web of Science, PubMed, Elsevier, Springer, and Wiley databases from inception till 2023. Keywords such as “*E. ulmoides* polysaccharides”, “*E. ulmoides* bark polysaccharides”, “*E. ulmoides* leaves polysaccharides”, and “*E. ulmoides* leaf polysaccharides” were used to collect the literature on extraction, purification, structural characteristics, bioactivities, applications and other aspects of polysaccharides derived from *E. ulmoides*. Meanwhile, duplicate and irrelevant literatures were excluded.

## 3 Preparation of EU polysaccharides

**FIGURE 1:**
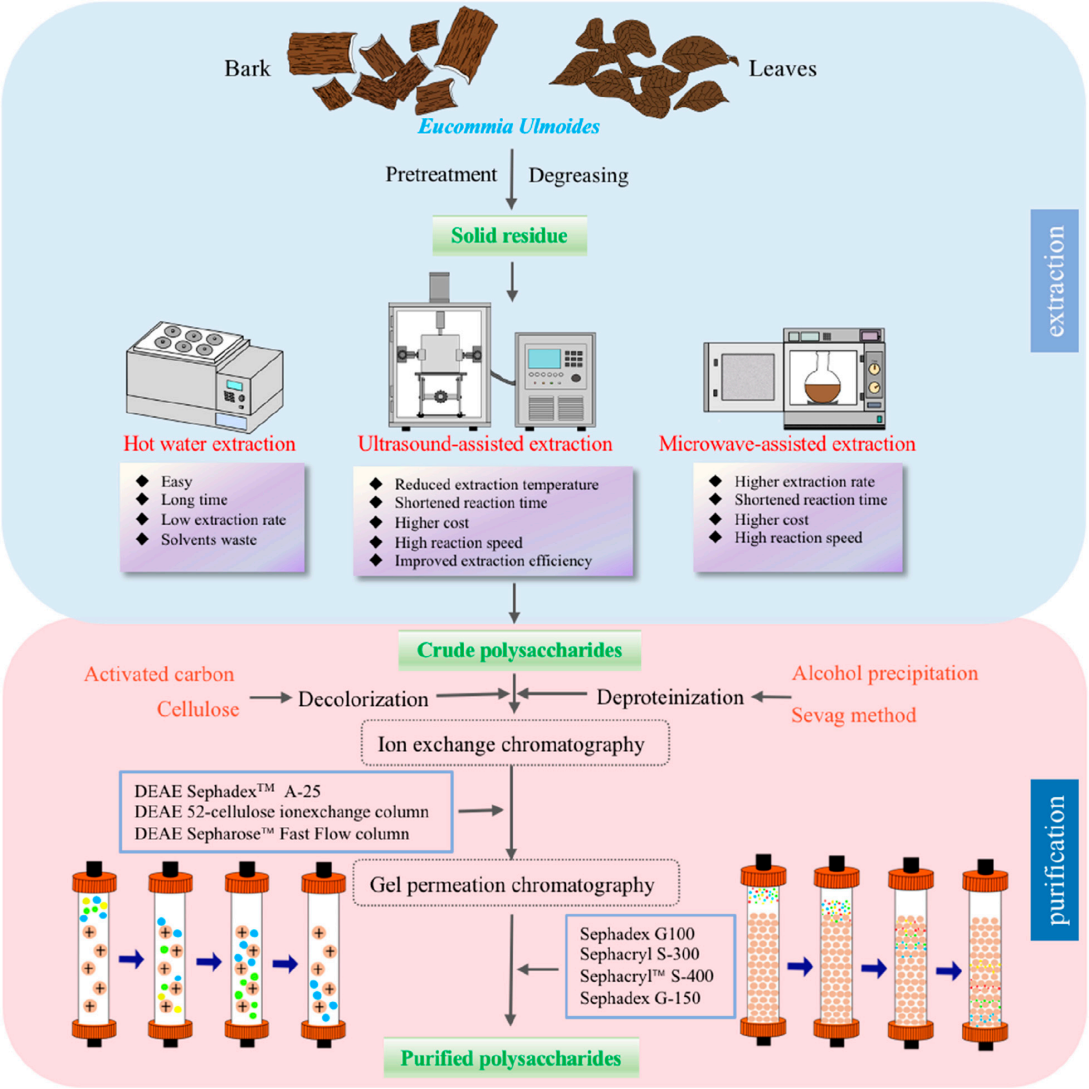
The flowchart of extraction methods and purification of *Eucommia ulmoides* polysaccharides.

Generally, the preparation methods for polysaccharides should be determined according to their composition and structure, and the selection of preparation methods should account for the chemical properties of components, extraction conditions, impurities and other factors (Sheng et al., 2021; Qin et al., 2022; Xu et al., 2022). Therefore, to efficiently and safely prepare polysaccharides without compromising their structural integrity, it is imperative to carefully select the appropriate preparation method. In recent years, technological and scientific advancements have improved the EU polysaccharide extraction and purification efficiencies (Zhu et al., 2009; Xia and Piao, 2010; Xu et al., 2018; Liu M. et al., 2020). Figure 1 briefly illustrates the preparation process of EU polysaccharides.

### 3.1 Extraction methods

The first step in studying natural plant polysaccharides is extraction, which is also the most important step in the isolation of bioactive substances (Shi, 2016; Mohan et al., 2020; Zeng et al., 2022). Specifically, polysaccharides with relatively high polarity are more soluble in water than ethanol (Li et al., 2021). Therefore, polysaccharides are normally extracted by the hot water extraction (HWE) method (Wang et al., 2019; Barbosa et al., 2020). In recent years, new methods with improved speed and efficiency have been developed to improve production efficiency or obtain higher yields (Liu et al., 2018), including alkali extraction (AE) (Zhu et al., 2016), microwave-assisted extraction (MAE) (Xu et al., 2018), and ultrasound-assisted extraction (UAE) (Liu M. et al., 2020). Table 1 summarizes the different extraction methods for EU polysaccharides.

**TABLE 1:** Different extraction methods of polysaccharides from *Eucommia ulmoides*.

Part	Source	Polysaccharide Name (fraction)	Extraction method	Conditions	Polysaccharide content	Yield	Reference
*E. ulmoides* bark	Purchased from Shanghai Hua-Yu Chinese Materia Medica Co. Ltd., China	EUPs(crude)	Hot water	-	Contained 69.80% of total carbohydrate and 28.86% of uronic acid	2.2%	Jiang et al. (2011)
*E. ulmoides* bark	-	EUP (crude)	Hot water	Time: 80min Solid and liquid ratio: 1:3 Extraction number 3	-	23.9%	Hong et al. (2013)
*E. ulmoides* bark	-	EUPS	Hot water	-	EUPS contained 97.4%, proteins content was 2.1%	-	Feng et al. (2016)
*E. ulmoides* bark	Purchased from China Pharmaceutical Corporation-Canton, China	EUP1	Hot water	-	Around 22.4% (EUP1), 9.2% (EUP2) and 37.5% (EUP3) of total EUP	-	Li et al. (2017)
*E. ulmoides* bark	China	Eucomman A	Hot water	-	-	0.22%	Gonda et al. (1990)
*E. ulmoides* bark	China	Eucomman B	Hot water	-	-	-	Tomoda et al. (1990)
*E. ulmoides* bark	Purchased from Huayu Materia Medica Co., Ltd, Shanghai, China	EWDS-1	Hot water	-	EWDS-1 contained 82.46% of total carbohydrate, 2.15% of uronic acid, 2.07% of sulfate, and 3.95% of protein	0.1‰	Zhu et al. (2008)
*E. ulmoides* bark	Purchased from Huayu Materia Medica Co., Ltd, Shanghai, China	EWDS-2	Hot water	-	Contained 92.32% of total carbohydrate, as well as 6.55% of protein	2.3%	Zhu et al. (2009)
*E. ulmoides* bark	Collected from the arboretum of Northwest Agricultural and Forestry University, Yangling, China	EUP1	Hot water	Temperature 60°C Time: 180min, liquid ratio: 1:20	-	5.99% of the original materials	Xu et al. (2015)
*E. ulmoides* bark	From a farm of the Northwest A&F University, Yangling, Shaanxi, China	FBWP	Hot water	-	Water-soluble polysaccharides (3.8%–8.6%)	4.13%	Zhu et al. (2016)
*E. ulmoides* bark	From a farm of the Northwest A&F University, Yangling, Shaanxi, China	FBAP	Alkali	-	Alkali-extractable polysaccharides (12.2%–26.1%)	12.19%	Zhu et al. (2016)
*E. ulmoides* bark	Collected from the arboretum of Northwest Agricultural and Forestry University, Yangling, China	EUP2	Ultrasound-assisted extraction	Room temperature Time: 60min, liquid ratio: 1:20	-	7.38% of the original materials	Xu et al. (2015)
*E. ulmoides* leaves	Collected from the arboretum of Northwest Agricultural and Forestry University, Yangling, China	EULP1	Hot water	Temperature 60°C Time: 180min, liquid ratio: 1:20	-	9.26% of the original materials	Xu et al. (2015)
*E. ulmoides* leaves	Obtained from SPH Zunyi Pharmaceutical Co.,Ltd. (Zunyi, China)	PsEUL	Hot water	Temperature 60°C Time: 120min, liquid ratio: 1:20	Total sugar content was 96.36%, ratio of polysaccharide to protein was determined to be 12:25	5.7%	Feng et al. (2021a)
*E. ulmoides* leaves	From a farm of the Northwest A&F University, Yangling, Shaanxi, China	LWPE	Hot water	-	Water-soluble polysaccharides (3.8%–8.6%)	5.03%	Zhu et al. (2016)
*E. ulmoides* leaves	From a farm of the Northwest A&F University, Yangling, Shaanxi, China	LAP	Alkali	-	Alkali-extractable polysaccharides (12.2%–26.1%)	26.06%	Zhu et al. (2016)
*E. ulmoides* leaves	Collected from the arboretum of Northwest Agricultural and Forestry University, Yangling, China	EULP2	Ultrasound-assisted extraction	Room temperature Time: 60min, liquid ratio: 1:20	-	10.51% of the original materials	Xu et al. (2015)
*E. ulmoides* leaves	Purchased from SPH Zunyi Pharmaceutical Co. Ltd (Zunyi, China)	PsEUL	Ultrasound-assisted extraction	Temperature 60°C Time: 120min, liquid ratio: 1:20	-	-	Feng et al. (2022)
*E. ulmoides* leaves	Collected from *E. ulmoides* research base of Chinese Academy of Forestry, China	Cp	Ultrasound-assisted extraction	Temperature 60°C Time: 80min, liquid ratio: 1:30, Power 200 W	164.95 mg/g	10.49%	Liu et al. (2020a)
*E. ulmoides* leaves	Harvested from the arboretum of Northwest A & F University, Yangling, China	EULP	Microwave-assisted extraction	Temperature 74°C Time: 15min, liquid ratio: 1:29	Carbohydrates contained 84.2%–89.5%	12.31%	Xu et al. (2018)
*E. ulmoides* leaves and bark	From a farm of the Northwest A&F University, Yangling, Shaanxi, China	LBWP	Hot water	-	Water-soluble polysaccharides (3.8%–8.6%)	4.54%	Zhu et al. (2016)
*E. ulmoides* leaves and bark	From a farm of the Northwest A&F University, Yangling, Shaanxi, China	LBAP	Alkali	-	Alkali-extractable polysaccharides (12.2%–26.1%)	16.07%	Zhu et al. (2016)

-: information was not available.

#### 3.1.1 Hot water extraction method

The hot water extraction process, which uses abundant and environmentally friendly water as the principal solvent, is the most traditional method of extracting EU polysaccharides, which includes decoction, soaking, and reflux (Khoza et al., 2014). During this process, extraction conditions, such as the temperature, duration, frequency, and solvent-drug ratio, all significantly affect the extraction yield of polysaccharides (Zhang et al., 2021). Typically, herbs are pretreated prior to extraction, and then the extraction operation is carried out (Zhu et al., 2008; Zhu et al., 2009). For instance. Zhu et al. (2008), Zhu et al., 2009) crushed EU bark (9 kg) into powder, degreased it with 95% ethanol, and extracted it with hot water to obtain insoluble parts; the yield was 2% of the dry matter. In a similar manner. Jiang et al. (2011) ground dried bark into fine particles, defatted it using 95% ethanol, and extracted the insoluble part with hot water, and the yield of crude EU polysaccharides was 2.2% of the dry matter. To improve the extraction rate, Hong et al. (Hong et al., 2013) used five volumes of distilled water to extract EU powder (500 g) at 100°C several times. Through contour plot and variance analysis, the optimal extraction process conditions were found to consist of an 80-min extraction time, a water-material ratio of 3, and extraction times of 3, and the yield reached 23.9%. In addition to crushing medicinal materials, some scholars cut them into pieces or strips and then extracted them. For example. Li et al. (2017) cut EU bark into strips and defatted it with 95% ethanol. They then boiled the bark in water three times for 3 h each to extract the necessary compounds. Moreover, Tomoda et al. (Gonda et al., 1990; Tomoda et al., 1990) extracted sliced bark (810 g) with hot water (8.1 L) under stirring for 30 min, and the yield was 0.22%. It follows that particle size significantly affects the extraction rate of EU polysaccharides (Li et al., 2023). The increased crushing degree can generate finer particles from the raw materials, leading to an initial increase in extraction yield due to a greater contact surface area, improved mass transfer rate, and lowered diffusional resistances (Gao et al., 2023).

Overall, hot or boiling water extraction is the most commonly adopted method in industrial and laboratory settings because it is inexpensive, environmentally friendly, and simple and minimizes damage to the polysaccharide structure (Hromádková et al., 2002; Zhang et al., 2020; Wang and Zhang, 2021; Jiang et al., 2022). However, its disadvantage lies in the prolonged extraction time, which leads to a slower extraction rate, and at the same time, the high solid‒liquid ratio leads to a waste of resources. Furthermore, high temperature can cause the destruction of the polysaccharide structure, thereby reducing its pharmacological activity (Hromádková et al., 2002; Xing et al., 2013; Yan et al., 2013). To compensate for these shortcomings, alternative extraction methods, including UAE, MAE and AE, have been developed (Ying et al., 2011). Microwave and ultrasound extraction methods have less impact on polysaccharide extraction based on particle size compared to hot water extraction. However, extraction can still affect the biological activity of polysaccharides, so multiple factors should be considered when choosing a method.

#### 3.1.2 Microwave-assisted extraction method

MAE has been widely used to extract bioactive compounds from natural materials. MAE technology can penetrate cell membranes and rupture cell walls, thereby releasing high-molecular weight (Mw) compounds such as polysaccharides into the solvent (Chen et al., 2017; Sharma et al., 2022). Briefly. Xu et al. (2018) mixed 2.0 g of the ground EU leaf sample with distilled water in a conical flask with a specific volume. The microwave generator was set to 74°C for 15 min for extraction. The resulting extracts were vacuum-filtered and concentrated at 40°C. Three volumes of ethanol (95%) were slowly added to the concentrated filtrate under continuous agitation. Through centrifugation and lyophilization, the precipitate was recovered, resulting in the isolation of the polysaccharide EULP-MAE with a yield of 12.31%. In addition, as seen in Table 1, significantly higher polysaccharide yields than with hot water extraction and UAE techniques were observed. Evidently, MAE of polysaccharides is an efficient method to increase yield (Lal et al., 2021). In contrast with the traditional method, MAE offers advantages such as a reduction in solvent consumption, a reduced extraction time, and an enhanced extraction yield (Zeng et al., 2012; Ekezie et al., 2017; Bagade and Patil, 2021). However, the drawbacks of this method are that the microwave extraction equipment is complex, and the extraction range is limited (Wen et al., 2018; Huang G. et al., 2021). Currently, MAE of EU polysaccharides is only used for *Eucommiae folium*, which may be due to the differences in composition and structure between *Eucommiae folium* and *Eucommiae cortex*, and the specific extraction conditions may need to be adjusted and optimized for *Eucommiae cortex*. In the future, it is necessary to further evaluate the use of microwave technology to extract polysaccharides from *Eucommiae cortex* to expand the application scope and value of this technology. Specifically, MAE equipment can be optimized to provide a more efficient, reliable, and operable microwave generator. New methods of MAE, such as cooperative MAE and adding extracts after extraction, can be studied to improve extraction efficiency and product purity.

#### 3.1.3 Ultrasound-assisted extraction method

UAE is also a new extraction technique that harnesses the high-frequency vibration of ultrasound and thermal effects within cavities to rupture cell membranes to increase solvent penetration into the tissue (Ebringerová and Hromádková, 2010; Liu et al., 2021). EU polysaccharides can also be extracted using this method. For instance. Xia and Piao. (2010) dried and crushed EU leaves and sieved them with 60 mesh. EU powder was immersed in petroleum ether, and degreasing was carried out by ultrasonic treatment at 30°C for 40 min. The solution was then mixed with 95% alcohol and sonicated at 80°C for 20 min to further remove impurities. Treated samples were immersed in water for 12–18 h, and UAE was performed. The optimal extraction conditions were determined to be an extraction temperature of 80°C, an extraction time of 40 min, a solid‒liquid ratio of 1:35, and two repeated extractions, and a polysaccharide extraction rate of 4.892%, and a polysaccharide purity of 86.76% were obtained. Furthermore, Liu et al. (Liu M. et al., 2020) dried EU leaves to constant weight in a thermostatically controlled blower at a temperature of 50°C. Then, the samples were crushed and filtered with a 60-mesh screen. Polysaccharides were extracted from EU leaves employing UAE technology. The four factors of extraction solution ratio (v/w), time (min), temperature (°C), and power (W) were optimized by orthogonal experiments to improve and enhance the extraction rate (%). Four independent variables were studied at three distinct levels (1, 2, and 3), and the supernatant obtained after centrifugation was the crude polysaccharide (Cp). The findings showed that the optimal extraction parameters encompassed an extraction liquid ratio of 30, an extraction time of 80 min, an extraction temperature of 60°C, an extraction power of 200 W, and the extraction yield increased from 4.82% to 10.49%. These results indicate that the UAE method has better extraction efficiency for EU polysaccharides than traditional methods such as the hot water extraction method (Gong et al., 2020). As a result, the extraction process and activity characteristics of EU polysaccharides is incomplete, and the comprehensive understanding and application of EU polysaccharides are limited. In future research, both EU leaf polysaccharides and EU bark polysaccharides should be studied to gain a thorough understanding of their extraction methods and properties for future applications.

Compared with the traditional hot water method, the extraction of polysaccharides can be enhanced by UAE, resulting in higher purity of extracted components at a lower temperature and reduced solvent usage (Hromádková et al., 2002; Zhang et al., 2009). However, this method is expensive and has not been widely adopted in industrial fields (Luft et al., 2021). Moreover, studies have shown that ultrasonic extraction can lead to polysaccharide degradation, molecular weight becomes smaller, thus affecting its biological activity (Li S. et al., 2022). The ultrasonic extraction of EU polysaccharides also has this problem, so solving this problem is very important for the development of EU polysaccharides in the future. With the ongoing evolution of polysaccharide extraction techniques, several novel methods have emerged, including supercritical fluid extraction, supercritical CO_2_ extraction, and ultrafiltration extraction. The exploration of these novel techniques can be employed to extract EU polysaccharides, effectively rectifying the shortcomings of the existing methodologies. Consequently, the necessity for further investigation and development of an optimized extraction technique for the isolation of EU polysaccharides cannot be ignored.

#### 3.1.4 Alkali extraction method

Studies have shown that extraction under alkaline conditions causes cell wall swelling, and then the cell wall of the plant is destroyed, causing it to release additional polysaccharides. Thus, a specific concentration of an alkali solution can be employed to extract cell wall-bound polysaccharides or intracellular polysaccharides, with the aim of enhancing the yield of polysaccharides (Sun Y. et al., 2022; Chen et al., 2022). A scholar used this method to extract EU polysaccharides. Xu et al. (2015) treated EU powder with 1 M NaOH aqueous solution for 3 h at 75°C using a solid‒liquid ratio of 1:20 g/mL. Subsequently, the base extraction filtrate was neutralized to pH 5.5 and concentrated to approximately 30 mL with 6 M HCl. Subsequently, the concentrate was removed with three volumes of 95% ethanol with vigorous stirring, filtered, washed twice with three volumes of 95% ethanol, and freeze-dried. Ultimately, the yield of polysaccharides extracted from different sources of EU ranged from 12.19% to 26.06% (Bai et al., 2022). Comparing hot water extraction and AE showed that the yield of AE was much higher than that of hot water extraction. It is known that AE has the advantages of short extraction times, mild reaction conditions, and high yields. Notably, the glycosidic bond and structure of polysaccharides will be destroyed by excess alkali concentrations, and polysaccharide degradation will occur (Nie and Xie, 2011; Huang and Huang, 2020). Future research could explore alternative reaction media, such as acidified solutions at specific pH levels, neutral solutions, or organic solvents, which may have varying effects on the extraction of cell wall polysaccharides, potentially leading to an increase in polysaccharide production. It is worth mentioning that in addition to obtaining polysaccharides by destroying cell walls by alkali extraction method, recently researchers found that plant polysaccharides can also be extracted by alkali precipitation method. Polysaccharide CGP-AP obtained by alkali precipitation from *Chaetomium globosum* has better anti-inflammatory and antioxidant activities (Wang S. et al., 2024), which suggests that we can study the extraction of polysaccharides from EU by alkali precipitation method in the future. It is of great significance to analyze whether the structure and biological activity of the polysaccharide obtained by alkali precipitation and alcohol precipitation are different, which will broaden the application range of EU polysaccharides.

In summary, the current research on polysaccharides extraction methods of EU is limited to some methods that have been widely used in polysaccharide extraction. With the development of biotechnology, the use of microbial fermentation to modify polysaccharides has become a research hotspot. Microbial fermentation can not only improve the bioavailability of polysaccharides, but also increase the extraction rate of polysaccharides to a certain extent (Wang G. et al., 2023). However, at present, there are few researches on the extraction of polysaccharides by microbial fermentation, and the extraction of EU polysaccharides by this method is in the blank stage. Therefore, the study on the extraction of polysaccharides by microbial fermentation is the focus of future research, which has positive significance to expand the application range of polysaccharides from EU and improve its commercial value. In addition, enzyme-assisted extraction of polysaccharides has been widely used in recent years. Compared with other extraction methods, the enzyme-assisted extraction method has the advantages of mild reaction conditions, easy deterioration of products, high extraction efficiency, low cost, environmental protection and energy saving. Through literature review, we found that there have been relevant studies on the extraction of chemical components of EU except polysaccharides by enzyme extraction method, which indicates that enzyme extraction method may be used for the extraction of polysaccharides. The further study of this method to extract polysaccharides from EU and whether the polysaccharides obtained have better biological activity will play an important role in the future development of related fields.

### 3.2 Isolation and purification methods

The crude polysaccharides contained more proteins than purified polysaccharides; therefore, in polysaccharide purification, it is often necessary to remove excessive proteins and peptides from the sample to ensure the accuracy of subsequent analysis. Several methods exist for protein elimination, with the Sevag and trichloroacetic acid methodologies being the most common (Long et al., 2020; Mohammed et al., 2020). After the treatment of the above steps, the protein content in the sample was effectively controlled, and the inorganic salts were then removed by dialysis, the preferred method used in most laboratories (Zhang C. et al., 2019). Additionally, for further purification and separation, column chromatography and fractional precipitation may be used (Tang and Huang, 2022). After the above steps are completed, polysaccharides can be purified by concentration and drying. See Table 2 for purification methods for EU polysaccharides.

**TABLE 2:** Purification, monosaccharide compositions, molecular weight, and possible structures of polysaccharides from *Eucommia ulmoides*.

Part	Compound name	Purification methods	Monosaccharide compositions	Molecular weight	Possible structures	Reference
*E. ulmoides* bark	EPs	Dialysis	Man, Rha, GalA, Glc, Gal, Xyl, Ara (1.13:2.18: 3.15:82.71:0.79:2.02:1.99)	0.63–251 kDa	-	Sun et al. (2022b)
*E. ulmoides* bark	EUPs(crude)	-	Ara, Gal, Glc, Rha (6.35:3.15:1.47:1)	-	-	Jiang et al. (2011)
*E. ulmoides* bark	EUP	-	Glc, Gal, Ara, Rha (53.9–63.2:10.6–16.1: 6.7–18.7: 2.0–2.1)	-	-	Xu et al. (2015)
*E. ulmoides* bark	EUPS	Seriatim, dialysis, DEAE Sephadex™ A-25	Glc, Fru, Man, Fucose, Gal, Ara (36.6:16.6:14.2: 15.7:9.5:7.4)	1146.32 kDa	A pyranose sugar, contained a *β*-type glycosidic bond	Feng et al. (2016)
*E. ulmoides* bark	EUP1	DEAE 52-cellulose ion-exchange column, gel-filtration column (Sephadex G100)	Rha, Ara, Gal, Man, Glc (4.6:8.6:6.1:1:2)	358.1 kDa	→3,4-rha–1→3-glc-1→, →4-Man–1→4-Glc-1→, →4-Glc–1→4-Glc-1→, →4-Glc–1→3-Glc-1→, →3-Glc–1→4, 3-Rha–1→, →3,4-Rha–1→3,6-Gal-1→, →3,6-Gal–1→3,6-Gal–1→6-Gal-1→, →6-Gal–1→6-Gal–1→3-Gal-1→, Man–1→3,6-Gal-1→	Li et al. (2017)
*E. ulmoides* bark	Eucomman A	DEAE Sephadex A-25	Ara, Gal, Glc, Rha, GalA (8:6:4:5:8)	6 kDa	*α*-L-Ara*f* 1→, *α*-D-Glc*p* 1→, →5 *α*-L-Ara*f* 1→, →4 *α*-D-Glc*p* 1→, →3 *α*-L-Ara*f* 1→, *α*-L-Rha*p* 1→, *β*-D-Glc*p* 1→, →2 *α*-L-Rha*p* 1→, →3 *β*-D-Glc*p* 1→, →4 *β*-D-Glc*p* 1→, →6 *β*-D-Glc*p* 1→, →4 *α*-D-Glc*p*A 1→	Gonda et al. (1990)
*E. ulmoides* bark	Eucomman B	DEAE-Sephadex A-25, Sephacryl S-300	Ara, Gal, Rha, GalA (10:5:24:24)	-	*α*-1,2-linkedl-rhamno-*α*-1,4-linkedd-galacturonan, *α*-1,5-Linkedl-arabinofuranose, *α*-1,3-linkedl-arabinopyranose, *β*-1,3- and *β*-1,4-linkedd-galactose, 2,4-branchedl-rhamnose and 3,4-branchedd-galacturonic acid	Tomoda et al. (1990)
*E. ulmoides* bark	EWDS-1	DEAE Sepharose™ Fast Flow column, Sephacryl™ S-400	Gal, Glc and Ara (2.1:1.0:0.9)	2000 kDa	(1,3,4,5-tetra-O-acetyl-2-O-methyl-arabinose (1,3,5-linked *α*-L-Araf),1,4,5,6-tetra-O-acetyl-2-O-methyl-galactose (1,4,6-linked *β*-D-Galp)), 1,5-linked and 1,3,5-linked *α*-L-Araf, 1,4-linked, 1,6-linked, and 1,4,6-linked *β*-D-Galp, 1,3-linked and 1,4-linked *β*-D-Glcp	Zhu et al. (2008)
*E. ulmoides* bark	EWDS-2	DEAE Sepharose™ Fast Flow column, Sephacryl™ S-400	Glc, Gal, Ara, and Rha (2.2:1.0:0.4:0.2)	1000–2000 kDa	1,3-linked, 1,4-linked, 1,2,6-linked, 1,3,6-linked Glc; 1,6-linked, 1,2,6-linked, 1,3,4-linked, 1,4,6-linked Gal; 1,5-linked, 1,3,5-linked Ara; terminal and 1,2,5-linked Rha	Zhu et al. (2009)
*E. ulmoides* bark	FBWP	Enzymatic hydrolysis	Glc, Gal, Ara (31.8–53.4:16.5–23.7:15.6–30.2)	17,540 g/mol	-	Zhu et al. (2016)
*E. ulmoides* bark	FBAP	Enzymatic hydrolysis	Ara, Xyl, Gal, Rha, Glc, Man GlcA (43.4–59.9:14.2–24.7:15.2–21.4:1.9–2.4:3.5–5.9: 0.8–1.0:2.9–3.7)	210.1 kDa	-	Zhu et al. (2016)
*E. ulmoides* leaves	PsEUL	Sephadex G-150 column, dialysis	Glc, Ara, Gal, Rha monohydrate (38.2–39.1:37.7:12.8: 11.8)	60–600 kDa	-	Feng et al. (2021b)
*E. ulmoides* leaves	LWPE	Enzymatic hydrolysis	Glc and Gal (77.5: 8.8)	49,870 g/mol	-	Zhu et al. (2016)
*E. ulmoides* leaves	LAP	Enzymatic hydrolysis	Ara, Xyl, Gal, Rha, Glc, Man GlcA (43.4–59.9: 14.2–24.7:15.2–21.4:1.9–2.4:3.5–5.9: 0.8–1.0:2.9–3.7)	62.1 kDa	-	Zhu et al. (2016)
*E. ulmoides* leaves	EULP	-	Glc, Gal, Rha (53.9–63.2:10.6–16.1:10.1–11.3)	-	-	Xu et al. (2015)
*E. ulmoides* leaves	EUP	-	Gul acid, ribose, Rha, Glucosamine, Glucuronic acid, Aminogalactose, Xyl (0.81:77.7: 4.44:2.07:1.15:7.76: 6.06)	3170 g/mol	-	Deng et al. (2019)
*E. ulmoides* leaves	PsEUL	D101 macroporous adsorption resin, DEAE SephadexTMA-25	-	-	A hexahedral and cubic structure, with a uniform distribution and relatively uniform size	Feng et al. (2022)
*E. ulmoides* leaves	Pp	Anion-exchange chromatography (DEAE-52 anion-exchange chromatography column), hierarchical alcohol sink method	-	-	-	Liu et al. (2020b)
*E. ulmoides* leaves	EULP	-	EULP-CHE, contained Rha, Ara, Gal, Glc, Xyl, Man, GlcA, and galacturonic acid (9:32:20:55:19:2:14:1) EULP-MAE contained Rha, Ara, Gal, Glc, Xyl, Man, GlcA, and GalA (7:4:6:14:1:2:3:1)	EULP-MAE 38,830 g/mol, EULP-CHE 12,055 g/mol	Belonged to a structure of *β*-type acidic heteropolysaccharides with a glucan group and highly branched degree	Xu et al. (2018)
*E. ulmoides* leaves and bark	LBAP	Enzymatic hydrolysis	Ara, Xyl, Gal, Rha, Glc, Man GlcA (43.4–59.9: 14.2–24.7: 15.2–21.4:1.9–2.4:3.5–5.9:0.8–1.0:2.9–3.7)	178.1 kDa	-	Zhu et al. (2016)
*E. ulmoides* leaves and bark	LBWP	Enzymatic hydrolysis	Glc, Gal, Ara (31.8–53.4:16.5–23.7:15.6–30.2)	27,890 g/mol	-	Zhu et al. (2016)

-: information was not available.

The first step in isolating and purifying polysaccharides is to remove the protein (Yang et al., 2009; Rajauria et al., 2021), for which there are many methods. For example. Zhu et al. (2009) added trichloroacetic acid to a hot water extract to precipitate proteins. The water fraction was subsequently dialyzed in tap water for a period of 3 days, and the polysaccharide was precipitated by the addition of four volumes of 95% ethanol. Following centrifugation, the precipitate was extensively washed with ethyl alcohol anhydride and subsequently lyophilized to yield a crude polysaccharide. In addition. Feng et al. (2021a) evaporated the filtrate using the Sevag method at low pressure. Similarly, Jiang et al. used the same method for protein removal (Jiang et al., 2011). After extracting polysaccharides in boiling water, the polysaccharides were precipitated overnight at 4°C and centrifuged utilizing chloroform/n-butanol at a ratio of 4:1 by volume. Subsequently, the collected water phase was dialyzed at a cut-off point of 8,000–14000 Da, and the process was followed by lyophilization to yield the crude extract (Li et al., 2017). Furthermore, Liu et al. (Li et al., 2015) fractionated the precipitate with 30%, 40%, 50%, 60%, 70%, and 80% ethanol concentrations at 4°C for 24 h, obtaining the polysaccharide precipitates by centrifugation. The Savag method exhibits mild reaction conditions, but its efficiency is limited (Shi, 2016). Following extraction, the protein is treated with hydrogen peroxide or activated carbon to achieve decolorization (Liu et al., 2020). Notably, the above methods may lead to the loss of polysaccharides when removing proteins, and there is the problem of confusion, so it is necessary to carry out multiple extraction and purification steps. In addition, the trichloroacetic acid method is suitable for samples with high polysaccharide content, while the Sevag method has limited efficiency in samples with high polysaccharide content, so it is necessary to choose according to the actual situation. Currently, enzymatic hydrolysis is also used to remove proteins. This method involves the use of enzymes to break proteins into soluble fragments, which are then removed from polysaccharides. This can reduce the precipitation or agglutination encountered in the extraction process of polysaccharides and at the same time can improve the purity and Mw determination results of polysaccharides. However, according to the literature, enzymatic hydrolysis has not been applied in the removal of proteins from EU polysaccharides and should be studied.

In the purification of polysaccharides from EU, column chromatography is often used after the removal of inorganic salts, including ion exchange columns (usually filled with DEAE) and gel filtration columns (including Sephacryl and Sephadex) (Zhu et al., 2009). In general, the initial purification of crude polysaccharides is typically carried out through anion-exchange column chromatography, followed by gel column chromatography for subsequent purification (Shi, 2016). For instance. Zhu et al. (2009) utilized a DEAE Sepharose Fast Flow column to fractionate crude polysaccharides. The polysaccharide was first washed with water and gradually treated with 0.4, 0.8, 1.2, and 2.0 M NaCl solution to yield five subfractions. Subsequently, Sephacryl S-400 was employed for repeated size-exclusion column chromatography. In addition, Tomoda et al. applied crude polysaccharide onto a DEAE-Sephadex A-25 column (Pharmacia), eluted it with water and (NH_4_)_2_CO_3_, collected the eluate, and then applied it to a Sephacryl S-300 column. After elution with the same buffer, the eluate was further processed using a Sephadex G-25 column, eluted with water, concentrated and lyophilized (Tomoda et al., 1990). Moreover. Li et al. (2017) applied crude polysaccharides in conjunction with a DEAE 52-cellulose ion-exchange column to extract distinct acidic polysaccharides. Later, gel filtration column (Sephadex G100) chromatography led to the isolation of pure polysaccharides, which included EUP1, EUP2, and EUP3. Currently, anion-exchange columns and gel filtration columns have been widely used for polysaccharide purification. However, importantly, these methods have slow flow rates and are time consuming, particularly for acidic polysaccharides (Shi, 2016). To solve this problem, high-performance liquid chromatography (HPLC) can be used instead of traditional column chromatography. To further improve the purification efficiency and speed up the purification and to provide a more efficient and accurate method for the research and application of polysaccharides.

Highly purified and uniform polysaccharides were successfully isolated and obtained through a meticulous series of separation and purification techniques, including protein removal, decolorization, and column chromatography. Nevertheless, these processes can be both intricate and time-consuming and may require the use of significant amounts of volatile organic solvents such as ethanol. The three-phase partitioning (TPP) system and ultrafiltration technology can compensate for these shortcomings. They also have the advantages of high separation efficiency, simple equipment requirements and no pollution. These two methods can be used in future research on the isolation and purification of EU polysaccharides. They can effectively improve the extraction efficiency and purity of polysaccharides, thereby providing more high-quality raw materials for EU polysaccharides for research and application in biology, medicine and other fields.

## 4 Physicochemical and structural features of EU polysaccharides

Polysaccharides are a class of biological macromolecular substances with intricate structures that play crucial roles in biochemical and biomechanical functions (Boddohi and Kipper, 2010; Grube et al., 2020). A variety of techniques have been used to study and identify the physicochemical and structural characteristics of polysaccharides. Commonly used techniques include high-performance ion chromatography (HPIC), HPLC, gas chromatography (GC), ultraviolet visible spectroscopy (UV), Fourier transform infrared spectroscopy (IR) and nuclear magnetic resonance spectroscopy (NMR) (Cui et al., 2020). Table 2 provides details of reported EU polysaccharides, including name, monosaccharide composition, Mw, structure, and relevant references.

### 4.1 Thermal stability

Owing to the necessity of industrial thermal sterilization, thermal stability as one of the physicochemical properties of polysaccharides is a key feature for their industrial application of biological materials (Nuerxiati et al., 2019). Additionally, practicality and usefulness of plant polysaccharides are directly correlated to their thermal stability to maintain the appearance, nutrition, and healthiness of food and medicine (Feng Y. et al., 2021). Deng et al. (2019) used Differential Scanning Calorimetry (DSC) and Thermo Gravimetry Analysis (TGA) to investigate the thermal stability of EU polysaccharides (EUP) and strontium EU polysaccharides (EUP-Sr). EUP with the incorporation of strontium increased the amount of adsorbed water and decreased the total weight losses. It is indicated that EUP-Sr was found to have better thermal stability than EUP due to a new complex formed with thermal-stable property. Sun et al. (2018) successfully prepared the nanocrystalline cellulose (NCC)/EU gum (EUG) nanocomposite films. However, the thermal stability of the incorporation of 2, 4, 6, and 8% NCC were decreased to varying degrees. Fortunately, among them the 4% NCC film exhibited the highest thermal stability. It suggests that NCC/EUG nanocomposite films are able to widely used in the future in packaging applications or dryland agriculture. Liu et al. (2023a) measured differential scanning calorimetry (DSC) curves to assess thermal stability of three components (EUP1, EUP2 and EUP3) purified from EU leaves. They all exhibited good thermal stability below 270 °C, but EUP1 was found to have the highest thermal stability. Differences among all the three in thermal stability might result from different molecular weight and monosaccharide composition. Physicochemical properties of polysaccharides are strongly associated with their biological effects (Meng et al., 2018), however, up to now a lack of knowledge persists concerning comprehensive studies on physicochemical properties of EU polysaccharides, such as rheological property, gelling property, and solubility.

### 4.2 Molecular weight (Mw)

Mw is a crucial index affecting the pharmacological activities of polysaccharides (Sun et al., 2012; Zhang J. et al., 2019). Recently, gel permeation chromatography (GPC) and high-performance gel permeation chromatography (HPGPC) have been employed to determine the Mw of polysaccharides from EU. As an example. Feng et al. (2016) extracted the polysaccharide EU polysaccharides from the bark of EU. These authors determined its Mw using GPC on a Sephadex G-100 column and established a calibration curve with a dextran standard. The result showed that the Mw of EU polysaccharides was 1146.32 kDa. In addition. Zhu et al. (2008), Zhu et al. (2009) isolated and refined two polysaccharides (designated EWDS-1 and EWDS-2) from *Eucommiae cortex* and subsequently determined their respective Mws using HPGPC. Their findings indicated that EWDS-1 had an average Mw of approximately 2000 kDa, while EWDS-2 had an average Mw ranging from 1000∼2000 kDa. Moreover. Xu et al. (2014), Xu et al. (2018) utilized GPC with a refraction index detector (RID) to determine the Mw of polysaccharides from *Eucommiae folium* with the assistance of microwave extraction. The polysaccharides extracted with microwave extraction had a higher Mw and greater polydispersity than those prepared without microwave extraction. Simultaneously. Xu et al. (2015) found that the relative Mw of polysaccharides extracted from leaves (21,410–91,800 g/mol) was generally higher than that extracted from bark (17,995–32,125 g/mol) by GPC and RID. In addition, the relatively high Mw also indicated that ultrasonic treatment did not significantly alter the macromolecular structure of EU polysaccharides but was conducive to the dissolution of macromolecular polysaccharides in water. It is clear that the Mw of polysaccharides varies with variable factors such as extraction and purification methods, solvents, raw materials and calibration methods (Prajapati et al., 2013; ShanChen et al., 2019; Liao et al., 2020), so these factors need to be considered during optimization and studied in depth. Second, the extraction and purification methods used by different researchers are quite different, making comparison of the obtained Mw data difficult. Studies have found that low-Mw polysaccharides have greater antioxidant activity than high-Mw polysaccharides due to the challenge high-Mw polysaccharides face in penetrating cell membranes (Qi et al., 2005; Zha et al., 2016). However, according to the EU polysaccharides reviewed in this paper, polysaccharides with a high Mw have strong antioxidant activity, and this difference needs to be further studied in the future.

### 4.3 Monosaccharide composition

It is well known that polysaccharides are formed by a few monosaccharides linked by glycosidic bonds. Many exciting studies have shown that the bioactivity of polysaccharides is intricately linked to the composition and proportion of individual monosaccharides; therefore, it is important to study the monosaccharide composition. In general, the first step to determine monosaccharide composition is acid hydrolysis, after which samples are analysed utilizing GC and HPLC (Yan et al., 2014; Ferreira et al., 2015; Cao et al., 2019; Fan et al., 2019). In this review, we found that most *Eucommia* polysaccharides contained glucose (Glc), galactose (Gal) and arabinose (Ara), but their contents differed. Zhu et al. (2009) determined the monosaccharide composition of EWDS-2 by GC analysis of alditol acetates, which consisted of Glc, Gal, Ara, and rhamnose (Rha) (2.2:1.0:0.4:0.2), accompanied by trace amounts of mannose (Man) and 6.55% protein. Additionally. Feng et al. (2016) hydrolysed EU polysaccharides with 2 M trifluoroacetic acid (TFA) at 100°C and then analysed the monosaccharide composition of the sample by GC. The monosaccharide components of EU polysaccharides were Glc, fructose (Fru), Man, fucose (Fuc), Gal and Ara (36.6:16.6: 14.2:15.7: 9.5%:7.4%). In addition. Xu et al. (2015) identified the polysaccharide components of EU by sulfuric acid hydrolysis and found that leaf polysaccharides mainly contained Glc, Gal and Rha, while bark polysaccharides mainly contained Gal, Glc and Ara. Similarly. Xu et al. (2018) also found that two polysaccharides from *Eucommiae folium* obtained by conventional heat-reflux extraction (CHE) and MAE contained similar monosaccharides, but their contents were significantly different. At the same time, it is worth noting that both EULP-CHE and EULP-MAE contain large amounts of Glc, 38.2% and 39.1%, respectively. This suggests that some *β*-glucan may be present in the isolated polysaccharide, indicating that microwave-assisted extraction may be beneficial to the release of *β*-glucan. Furthermore. Zhu et al. (2016) found that water-soluble EU polysaccharides were primarily composed of Glc, while alkali-extracted polysaccharides were mainly composed of Ara. These results suggest that EU polysaccharides obtained from various plant parts and through various extraction processes have different chemical compositions, which can impact the medicinal properties and alter chemical structures (Huang et al., 2019; Wang et al., 2021; Wu et al., 2021). According to the study results of Xu et al., the antioxidant activity of leaf polysaccharides was higher than that of bark polysaccharides, which may be caused by the stronger antioxidant activity of Rha than of Ara, regardless of content. This indicates that monosaccharide composition may play a potentially crucial role in antioxidant activity. Further studies on the composition and proportion of monosaccharides in polysaccharides obtained from different parts and extraction processes are needed to clarify their biological activities. In addition, although the relationship between the biological activity of polysaccharides and the composition and proportion of monosaccharides has been studied, the mechanism by which the composition and proportion of monosaccharides affect biological activity has not been discussed in detail. Moreover, the biological activity of polysaccharides is also closely related to other components, so the existence and influence of these components need to be further studied. Investigating the interaction between the biological activity of polysaccharides and other components provides a scientific basis for the further development and utilization of polysaccharides.

### 4.4 Structural features

#### 4.4.1 Functional groups

Growing evidence has proved that a variety of polysaccharides with specific functional groups, such as acetyl groups, sulfate groups and phosphate groups, whether by chemical modifications or derived from natural resources possess better bioactivities than those that are naturally occurring without these functional groups (Song et al., 2015).

Acetyl groups of polysaccharides play a key role in their immunostimulatory activities (Ferreira et al., 2015). Fourier transformed infrared (FT-IR) spectroscopy is an available tool for identifying the functional groups of polysaccharides (Zhao et al., 2020). Liu et al. (2023b) used FT-IR spectroscopy to characterize the functional groups of three polysaccharide fractions derived from EU eluted by a column of DEAE-52 cellulose. The results showed that these three fractions all had an absorption peak at 1262 cm^−1^, suggesting that they contained acetyl groups. Likewise. Xu et al. (2018) indicated that under microwave irradiation decrease of acetyl groups linked to the polysaccharides were produced, and some acetyl groups attached to the polysaccharides derived from EU were confirmed by the presence of the weak peak at 1238 cm^−1^. Besides. Xu et al. (2015) reported the presence of some acetyl groups attached to the polysaccharides, particularly in the fraction isolated from EU leaves. This was due to the presence of the two weak absorbances at 1715 and 1240 cm^−1^. Nuclear magnetic resonance (NMR) spectroscopy is another important tool to obtain functional groups characteristic of polysaccharides (Huang et al., 2020). In ^1^H-NMR spectrum of the polysaccharide derived from EU, Liu et al. confirmed the presence of the acetyl group by the signal at δH1.9–2.3 ppm corresponded. This result was consistent with that of the FT-IR spectrum analysis mentioned above (Liu et al., 2023a).

Additionally, some studies have shown that the sulfate-group content of polysaccharides is usually associated with their biological activities, especially antitumor and anticoagulant activities (Wang et al., 2018; Li et al., 2020; Li T. et al., 2022). Polysaccharides with higher sulfate group content exhibit such higher bioactivities. Zhu et al. (2009) measured the sulfate content of the polysaccharide EWDS-2 with anti-complementary activity derived from EU using a modification of BaCl_2_ turbidimetric method. The result indicated that EWDS-2 was characterized with 2.82% sulfate content. It might be consequently proved right that the sulfate group of polysaccharides was found to be required for the exhibition of their anti-complementary activity.

In the present study, however, the mechanistic details of biological activities of polysaccharide functional groups derived from UC was not deeply discussed. Their structure-activity relationships with regard to functional groups remain undetermined.

#### 4.4.2 Glycosidic bond

The polysaccharide’s overall structure is influenced depending not only upon the monosaccharide composition/molecular weights, but also upon the other factors, especially the glycosidic bonds (Hou et al., 2021). To analyze the polysaccharide structure in more detail in glycosidic bonds, it is necessary to clarify type of glycosides linkage and branching of polysaccharides (Zou et al., 2020). The glycosidic linkage and link manner of polysaccharides derived from EU were investigated by complete or partial acid hydrolysis, methylation, together with GC-MS, IR and NMR spectra analysis.

**FIGURE 2:**
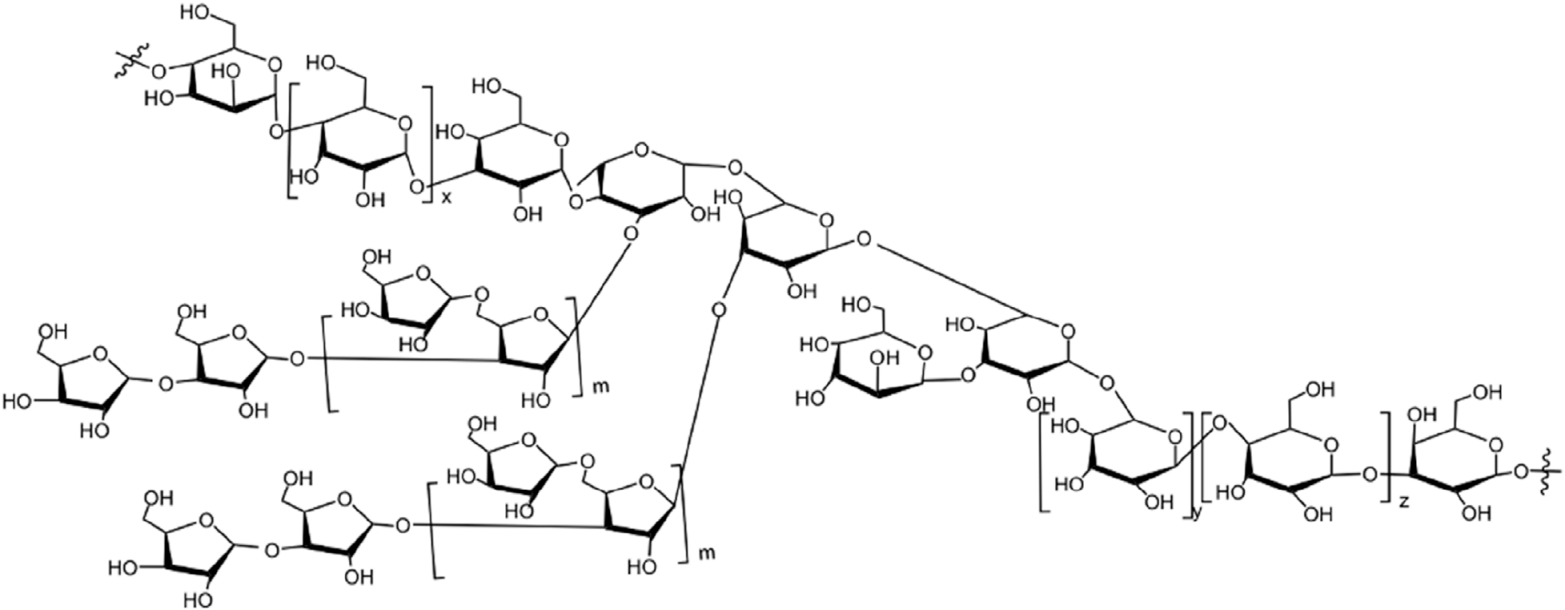
The hypothetical structure of EUP1.

Zhu et al. (2008), Zhu et al. (2009) obtained a homogeneous protein-bound polysaccharides EWDS-1 and EWDS-2 from the *Eucommiae cortex*. Through methylation analysis and NMR, the structure of their residues was deduced. EWDS-1 was a complex branched polysaccharide with an anti-complementary effect; the backbone was composed of Gal and Glc residues arranged in a pyranosidic form, while the side chains were enriched with Ara residues in the furanosyl configuration. Moreover, there were terminal, 1,5-linked and 1,3,5-linked *α*-L-Araf; terminal, 1,3-linked and 1,4-linked *β*-D-Glcp; and terminal, 1,4-linked, 1,6-linked, and 1,4,6-linked *β*-D-Galp. The main linkages of the residues of EWDS-2 that was also anti-complementary active include terminal, 1,6-linked, 1,2,6-linked, 1,3,4-linked, 1,4,6-linked Gal; 1,3-linked, 1,4-linked, 1,2,6-linked, and 1,3,6-linked Glc; 1,5-linked and 1,3,5-linked Ara; and terminal and 1,2,5-linked Rha. Similarly. Li et al. (2017) extracted a polysaccharide from EU bark, EUP1, which was characterized by methylation and NMR; the following linkages were identified: →3,4-Rha–1→3-Glc-1→, →4-Man–1→4-Glc-1→, →4-Glc–1→4-Glc-1→, →4-Glc–1→3-Glc-1→, →3-Glc–1→4,3-Rha–1→, →3,4-Rha–1→3,6-Gal-1→, →3,6-Gal–1→3,6-Gal–1→6-Gal-1→, →6-Gal–1→6-Gal–1→3-Gal-1→, and Man–1→3,6-Gal-1→. The possible chemical structure of EUP1 is shown in Figure 2. In addition, Tomoda et al. (Tomoda et al., 1990) found a glycan, eucomman B in the hot water extract of the bark of EU, and chemical and spectral studies have shown that this glycan predominantly constituted the characteristic structural subunits of *α*-1,2-linked L-Rha, *α*-1,4-linked D-galacturonan, *α*-1,5-linked L-arabinofuranose, α-1,3-linked L-arabinopyranose, β-1,3- and β-1,4-linked D-Gal, 2,4-branched L-Rha, and 3,4-branched D-galacturonic acid residues. This data shows that most polysaccharides obtained from EU are 1,3-linked, 1,4-linked, and 1,3,6-linked polysaccharides, but there are many different linkage sites and fragments, resulting in different chemical structures, which could be due to species differences and varying growth environments of EU, leading to different EU polysaccharide biological activities (Qiu et al., 2022). EU polysaccharides exhibit diverse types of glycosidic bonds and functional groups.

#### 4.4.3 Spatial structure

Besides functional groups, glycosidic bond, et al., spatial structure is also one of the important parameters of plant polysaccharides. And spatial structure or conformation of polysaccharides was found to be decisive for the biological activity (Kolsi et al., 2016; Hou et al., 2021). Increasing studies focus on exploring the advanced structure of polysaccharides to elucidate the relationship between their spatial structure and biological function (Lin and Huang, 2022). Morphologically, being soluble in aqueous solution, polysaccharide molecules may present diverse conformations, such as random coil, rod-lone, sphere-like shapes, and helical structures (Lin and Huang, 2022; Fu et al., 2024).

Deng et al. (2019) compared the particle sizes of EUP and EUP-Sr by scanning electron microscopy (SEM). Interestingly, EUP-Sr was found to have a more uniform and smaller particle size than EUP. This was probably because strontium incorporation had proved useful for optimization of the disordered structure of EUP. Deng et al. also observed the morphologies of EUP and EUP-Sr by atomic force microscope (AFM). It could be observed from the AFM images that height distributions of both EUP and EUP-Sr were fairly narrow with spherical morphology. But there was a slightly difference between the two by height distribution analysis. Strontium incorporation may cause the smaller particle size of EUP-Sr, resulting in the aggregation of the molecular chain in EUP. In addition to spherical ones, EUPs derived from EU were also found to display a spongy appearance by SEM 500-fold, 3000-fold and 5000-fold amplification, presenting a rough surface with pores and crevices (Feng et al., 2016). Compared with conventional SEM, field emission scanning electron microscopy (FE-SEM) observations have several advantages, including providing a clearer picture with 3–5 times better resolution (Das et al., 2022). Liu et al. (2023b) used FE-SEM at 10 K×multiples to observe the surface microstructures of three polysaccharide components purified from EU leaves. The three resulting component structures presented completely different surface morphologies. EUP1, EUP2 and EUP3 were honeycomb-shaped, rod-shaped and fake structure with smooth surface, respectively. These differences could be related to their presented different aggregation and bonding strength.

Overall, the available literature on information on the spatial structures of EU polysaccharides is quite inadequate. Due to complex structures of polysaccharides, it is very difficult to fully characterize spatial structures or conformation. Therefore, more attention should be paid to novel and more effective techniques. More details obtained in spatial structure will subsequently help reveal some structure-activity relationship of EU polysaccharides in the future.

## 5 Biological activities of EU polysaccharides

**FIGURE 3:**
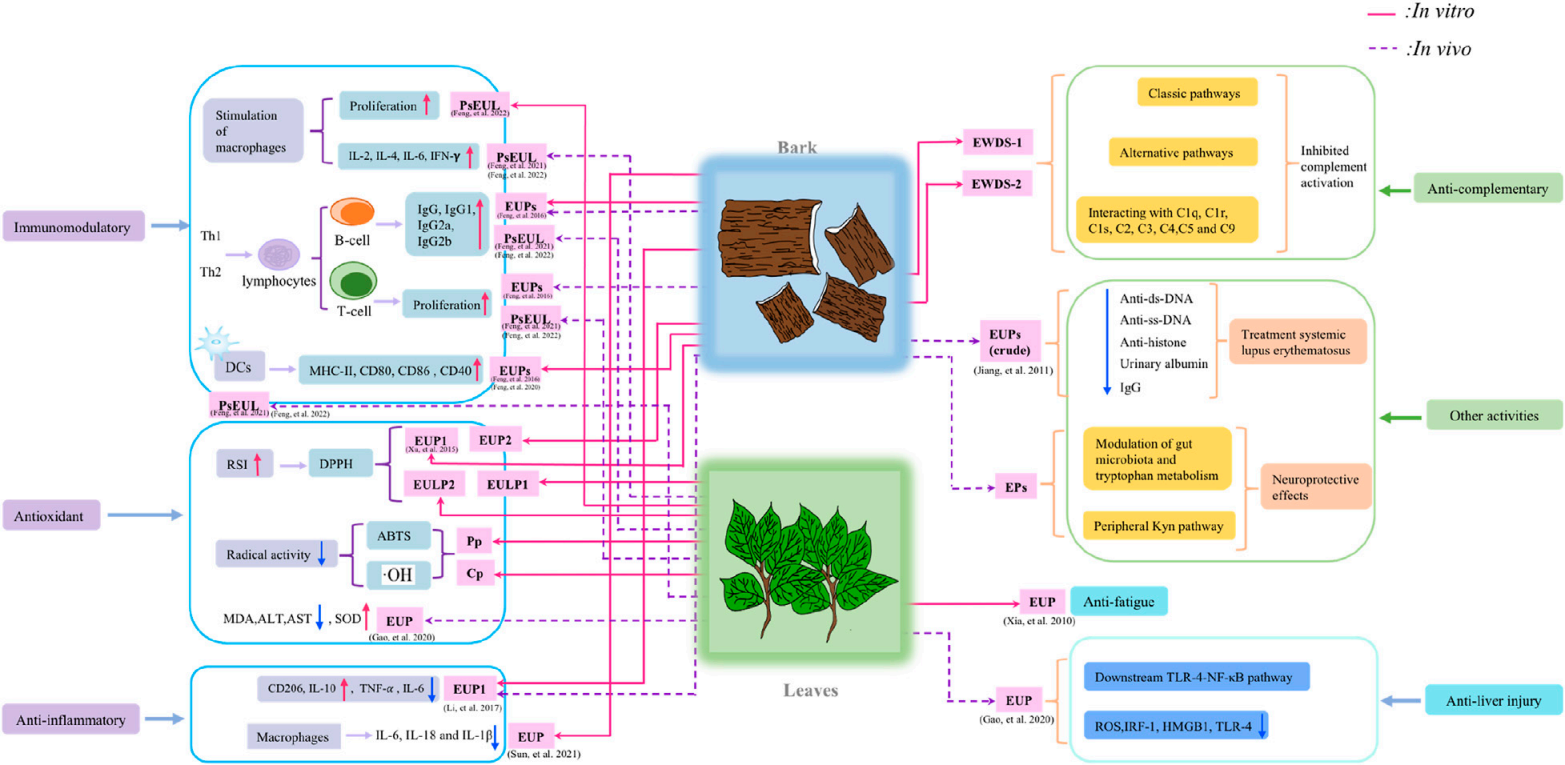
Biological activities of polysaccharides from *Eucommia ulmoides*.

EU is a valuable Chinese herb for treating diseases, and EU polysaccharides are important active ingredients due to their pharmacological function. The bioactivity of EU polysaccharides has been studied extensively *in vivo* and *in vitro*. Numerous studies have proven that EU polysaccharides have a wide range of biological activities, including immunomodulatory (Feng et al., 2016), antioxidant (Hong et al., 2013), anti-inflammatory (Sun et al., 2021), anticomplementary (Zhu et al., 2008), antifatigue (Xia and Piao, 2010), and hepatoprotective (Gao et al., 2020) activities. The comprehensive bioactivity information of EU polysaccharides is shown in Figure 3; Table 3.

**TABLE 3:** Summary of pharmacological activities of polysaccharides from *Eucommia ulmoides* and their applications or prospects.

Pharmacological activities	Name	Source	Types	Models/Testing subjects	Doses/duration	Effects/mechanisms	Applications/Prospects	Reference
Neuroprotective effects	EPs	*E. ulmoides* bark	*In vivo*	OD-induced obesity-related symptoms mice model	400 mg/kg	*Escherichia coli*, LPS, peripheral Kyn pathway, QA and glutamic acid↓	Mitigate obesogenic diet-induced cognitive and social dysfunction	Sun et al. (2022b)
A beneficial effect on systemic lupus erythematosus	EUPs(crude)	*E. ulmoides* bark	*In vivo*	Systemic lupus erythematosus-like syndrome induced by *campylobacter jejuni* in BALB/c mice model	15, 30 mg/kg/day	Anti-ds-DNA, anti-ss-DNA, anti-histone antibodies levels, IgG production, urinary albumin↓	New agent for the treatment of autoimmune disease	Jiang et al. (2011)
Antioxidant activity	EUP1	*E. ulmoides* bark	*In vitro*	The 2,2-diphenyl-1-picryl-hydrazyl (DPPH) radical	0.1, 0.2, 0.3, 0.4, 0.6, 0.8, 1.0, and1.2 mg/mL	The scavenging activity was 56.3%, RSI↑	Preparing nutritional health foods	Xu et al. (2015)
	EUP2	*E. ulmoides* bark	*In vitro*	The 2,2-diphenyl-1-picryl-hydrazyl (DPPH) radical	0.1, 0.2, 0.3, 0.4, 0.6, 0.8, 1.0, and1.2 mg/mL	The scavenging activity was 70.9%, RSI↑	Preparing nutritional health foods	Xu et al. (2015)
	EULP1	*E. ulmoides* leaves	*In vitro*	The 2,2-diphenyl-1-picryl-hydrazyl (DPPH) radical	0.1, 0.2, 0.3, 0.4, 0.6, 0.8, 1.0, and1.2 mg/mL	The scavenging activity was 89.6%, RSI↑	Preparing nutritional health foods	Xu et al. (2015)
	EULP2	*E. ulmoides* leaves	*In vitro*	The 2,2-diphenyl-1-picryl-hydrazyl (DPPH) radical	0.1, 0.2, 0.3, 0.4, 0.6, 0.8, 1.0, and1.2 mg/mL	The scavenging activity was 96.7%, RSI↑	Preparing nutritional health foods	Xu et al. (2015)
	Pp	*E. ulmoides* leaves	*In vitro*	DPPH radical, OH radical, ABTS radical	0.04 mg/mL to 0.18 mg/mL	Scavenging activity↑	Be applied as potential antioxidant	Liu et al. (2020b)
	Cp(crude)	*E. ulmoides* leaves	*In vitro*	DPPH radical, OH radical, ABTS radical	0.04 mg/mL to 0.18 mg/mL	Scavenging activity↑	Be applied as potential antioxidant	Liu et al. (2020b)
	EULP	*E. ulmoides* leaves	*In vitro*	DPPH radical	0.1–2.0 mg/mL	DPPH radical scavenging index (0.87–1.22) ↑	Act as a favorable antioxidant in functional food	Xu et al. (2018)
	EUP	*E. ulmoides* leaves	*In vitro*	Liver I/R model in rats	320 mg/kg, 160 mg/kg, 80 mg/kg for 10 days	SOD↑ ALT, AST and MDA↓	May be a promising drug in liver surgery to prevent HIRI	Gao et al. (2020)
Immunomodulatory effects	EUPS	*E. ulmoides* bark	*In vitro*	Mice splenocyte cell, mice bone marrow cells	100 μL for 68 h	MHC I/II, CD80, CD40, CD86, lymphocyte proliferation, IL-4 and IFN-γ↑	A strong immunostimulant, potential adjuvant for vaccine design	Feng et al. (2016)
	EUPS	*E. ulmoides* bark	*In vitro*	DC cells	5, 10, 20, 40, 80, 160, 320, 640, 1280 μg/mL for a further 24 h	IgG, IgG isotypes levels and maturation of DCs↑	Immune-enhancing agent and antigen delivery system	Feng et al. (2020a)
	PsEUL	*E. ulmoides* leaves	*In vitro*	RAW264.7	200 μg/mL for a further 24 h	Macrophages proliferation and phagocytosis↑	-	Feng et al. (2021a); Feng et al. (2022b)
	EUPS	*E. ulmoides* bark	*In vitro*	Females ICR mice	100 μg for 14 days	Splenocyte proliferation, NK cell and CTL cyto-toxicity↑	Immune-enhancing agent and antigen delivery system	Feng et al. (2020a)
	EUPS	*E. ulmoides* bark	*In vitro*	Female ICR mice	0.5 mg for 14 days	IgG, IgG1, IgG2a, IgG2b and T cell proliferation↑	A strong immunostimulant, potential adjuvant for vaccine design	Feng et al. (2016)
	PsEUL	*E. ulmoides* leaves	*In vivo*	Female ICR mice	500 μg/mL for 7 days	IgG, IgG1, IgG2a, IgG2b, IL-2, IL-4, IL-6, IFN-γ, T cells and DCs ↑	Developing natural polysaccharide-conjugated antigen nano delivery systems	Feng et al. (2021a); Feng et al. (2022)
Anti-inflammatory activity	EUP1	*E. ulmoides* bark	*In vitro*	Raw 264.7 cells	10 μg/mL, 25 μg/mL,50 μg/mL for 18 h	IL-10↑ IL-1β, TNF-α and IL-6↓	A valuable candidate for further development into an anti-septic therapeutic agent	Li et al. (2017)
	EUP	*E. ulmoides* bark	*In vitro*	RAW 264.7 cell	10,50,100,200 μg/mL for 1,3,5 days	BMP-6, Arg-1 and TGF-β ↑ IL-6, IL-18 and IL-1β, M1 polarized macrophages↓	Treatment for osteoarthritis	Sun et al. (2021)
	EUP-Sr	*E. ulmoides* bark	*In vitro*	RAW 264.7	10, 50, 100, 500 μg/mL for 1 and 3 days	Osteogenesis and inhibits osteoclastogenesis↑	Create a positive prodegenerative environment for skeleton tissue engineering	Deng et al. (2019)
	EUP1	*E. ulmoides* bark	*In vivo*	LPS-induced sepsis model in mice	10 mg/kg	CD206, IL-10↑ TNF-α↓	A valuable candidate for further development into an anti-septic therapeutic agent	Li et al. (2017)
Anti-complementary activity	EWDS-1	*E. ulmoides* bark	*In vitro*	Sheep erythrocytes, Guinea pigs, Rabbit erythrocytes, NHS	100,150,200 μL	Classical pathway, alternative pathway and spontaneous activation of NHS↓	Promising benefits in treatment of the complement associated diseases	Zhu et al. (2008)
	EWDS-2	*E. ulmoides* bark	*In vitro*	NHS, Guinea pigs, Sheep erythrocytes, Rabbit erythrocytes	100,150,200 μL	Classical pathway, alternative pathway and spontaneous activation of NHS↓	Be valuable for the treatment of diseases associated with the excessive activation of the complement system	Zhu et al. (2009)
Anti-fatigue	EUP	*E. ulmoides* leaves	*In vitro*	Gastrocnemius was isola-ted from toad	0.01, 0.05, 0.25 mg/mL	The maximum contraction time of toad muscle and the muscular fatigue↓	Develop a new medicine for the preventing and remedying of myocardial ischemia reperfusion induced injury	Xia and Piao (2010)
Anti-liver injury	EUP	*E. ulmoides* leaves	*In vivo*	Liver I/R model rat serum	320 mg/kg, 160 mg/kg, 80 mg/kg for 10 days	IRF-1, HMGB1↑ TNF-α, IL-1β, ROS, TLR-4, MyD88, P-p65, P-IKB-αproteins and TLR-4-NFκB pathway↓	May be a promising drug in liver surgery to prevent HIRI	Gao et al. (2020)

↑: improve or promote.

↓: inhibit or reduce.

-: information was not available.

### 5.1 Immunostimulatory activity

Numerous studies have demonstrated that plant polysaccharides exert various effects on immune regulation but cause almost no adverse reactions (Chen et al., 2021b; Zhang et al., 2021). This phenomenon is mainly achieved through their impacts on the immune system, such as the activation of macrophages and dendritic cells (DCs). Macrophages have multiple functions, serving as crucial components of the immune system by actively participating in both specific and nonspecific immune responses (Artyomov et al., 2016). DCs are closely related to the development of tumors and are the most powerful antigen-presenting cells in the immune system (Kim et al., 2009). The activity of macrophages and DCs is a key indicator of immune activation and an important part of immune effect research (Yuan et al., 2015). Most scholars choose to detect DCs and macrophages to determine immune activity. Sun et al. (2021) found that EU polysaccharides, at concentrations of 50 μg/mL and 100 μg/mL, exhibited potent stimulatory effects on macrophage proliferation. This effect was accompanied by a reduction in M1-polarized macrophages and an accumulation of M2-polarized macrophages. Additionally. Feng et al. (2016) performed *in vitro* and *in vivo* experiments. The *in vitro* experiments showed that EU polysaccharides (1.2–75 μg/mL) induced DC maturity and not only increased MHC I/II, CD80, CD86 and CD40 expression but also significantly stimulated lymphocyte proliferation and effectively augmented the production of IL-4 and IFN-*γ* cytokines. During the *in vivo* study, it was found that EU polysaccharides could increase T-cell proliferation and production of IgG, IgG1, IgG2a, and IgG2b antibody titres. In summary, EU polysaccharides are strong immunostimulants that can potentially enhance the efficacy of vaccines.

Notably, modified polysaccharides enhance immunomodulatory activity. To develop a new bone immunomodulatory biomaterial based on EU polysaccharides. Deng et al. (2019) successfully synthesized strontium-EU polysaccharide conjugates (EUP-Sr). The results of the CCK8 assay showed that EUP-Sr exerted a positive influence on the proliferation of macrophages and could inhibit the production of inflammatory factors and osteoclasts. The aim of the study was to enhance the adjuvant activity of polysaccharides isolated from *Eucommiae folium* (PsEUL) in potentiating an efficient immune response against ovalbumin (OVA). Feng et al. accomplished conjugation of PsEUL to OVA by employing the one-ethyl-3-(3-dimethylaminopropyl) carbodiimide hydrochloride (EDC) method. Experimental *in vitro* and *in vivo* studies demonstrated that the PsEUL-OVA/Cubs formulation elicited robust immune responses through the enhancement of phagocytic activity in DCs and macrophages, thereby enhancing antigen presentation efficiency (Feng et al., 2021; Feng et al., 2022). Furthermore, Feng et al. (Feng et al., 2020) ingeniously encased OVA and EU polysaccharides within long-circling nanoliposomes and conjugated them with an antibody targeting the DEC-205 receptor to develop a groundbreaking nanoliposome formulation known as anti-DEC-205-EUPS-OVA-LPSM. This formulation effectively served as a means of antigen delivery, thereby enhancing both cellular and humoral immune responses by stimulating the maturation of DCs. These studies lay the foundation for the development of novel adjuvant-antigen delivery systems. These findings highlight the potential usefulness of EU polysaccharides as a natural active ingredient to enhance and regulate human immune function.

Collectively, the immunomodulatory activity of EU polysaccharides controls and regulates the balance of the immune response, promoting the release of immunoreactive substances and enhancing immune cell function. It is clear that EU polysaccharides show potential as novel immunomodulators with therapeutic potential for a broad spectrum of diseases. However, most current studies are limited to *in vitro* studies utilizing a limited selection of cell lines, necessitating further *in vivo* and clinical investigations to fully evaluate their therapeutic potential. For the study of macrophages and DCs, we focused on their quantitative changes and antigen presentation ability and ignored their specific roles in the immune response. Their mechanism of action in the specific immune response should be further explored in combined *in vitro* and *in vivo* experiments. Consistent focus is needed to understand how these factors interact in order to effectively change polysaccharides and achieve the best results.

### 5.2 Antioxidant activity

Oxidative stress refers to the detrimental impact resulting from the excessive generation of ROS in the human body, which is associated with the occurrence of a myriad of ailments, including cancer, diabetes mellitus, and atherosclerosis (Ismail Iid et al., 2020; Chen et al., 2021). Antioxidants can prevent and reduce damage to the body to a certain extent. The current study found that natural plants are valuable sources of novel antioxidants, and their main active components are polysaccharides. Usually, antioxidant activities are evaluated through the utilization of various free radical assays, including DPPH, hydroxyl, and ABTS radicals *in vitro* (Luan et al., 2021). For example, Hong et al. (Hong et al., 2013) found that EU polysaccharide extracts reversed IR-induced decreases in superoxide dismutase (SOD), catalase (CAT), glutathione peroxidase (GSH-Px), and glutathione reductase (GR) activities in the kidney in a dose-dependent manner. These results suggest that EU polysaccharides may play a protective role in rabbit kidney oxidative injury through their free radical scavenging activities. In addition. Liu et al., (2020a) obtained EU leaf polysaccharides by UAE. Through meticulous power tests, the DPPH test, OH test and ABTS test, it was found that EU polysaccharides had notable DPPH, OH and ABTS radical scavenging activities. Notably, the 50% inhibitory concentration (IC_50_) values of EU polysaccharides for scavenging DPPH radicals in Cp (crude) and Pp (pure) were found to be 0.005 mg/mL and 0.011 mg/mL, respectively. additionally. Xu et al. (2015) used UAE of polysaccharides from EU bark and leaves. The findings revealed that ultrasonic treatment improved the yield and selectivity of polysaccharide antioxidants. Furthermore, the antioxidant activity of leaves proved to be significantly higher than that of the bark. EU polysaccharides can act as potential antioxidants, and there is a strong correlation between antioxidant activity and the content of bioactive compounds. Although various studies have proven the antioxidant actions of EU polysaccharides *in vitro*, there have been few *in vivo* studies, which need further investigation. The potential molecular mechanism of the antioxidant effect of EU polysaccharides is unclear, and it is worthwhile to explore the underlying mechanisms responsible for this effect.

### 5.3 Anti-inflammatory activity

Inflammation is a crucial defensive mechanism of the body’s immune system, triggered by inflammatory agents or physical damage to the tissue. According to existing research results, the aetiology of numerous ailments stems from inflammatory processes, such as obesity, hypertension and cancer (Fang et al., 2020; Li and Huang, 2021). Among natural products, polysaccharides have immense potential as anti-inflammatory drugs. In recent years, the safety and anti-inflammatory activity of plant polysaccharides have attracted increasing attention from researchers worldwide (Hou et al., 2021). It was found that EU polysaccharides had anti-inflammatory activity. For instance. Sun et al. (2021) found that EU polysaccharides had a positive effect on osteoarthritis. *In vitro*, inhibition of the expression of inflammation-related genes, such as IL-6, IL-18, and IL-1*β*, was observed following exposure to EU polysaccharides, while the expression of bone and chondrogenic genes, such as BMP-6, Arg-1, and TGF-*β*, was enhanced. *In vivo*, the destruction of articular cartilage is reduced. Additionally, Li et al. (2017) reported that the polysaccharide EUP1 from EU bark promoted the expression of CD206, a pivotal proinflammatory cytokine, and IL-10 in Raw 264.7 cells. These results indicate that EUP1 can effectively suppress the expression of major inflammatory factors, reduce lung injury, and augment the survival rate of animals. Furthermore. Deng et al. (2019) found that EUP-Sr could enhance the expression of osteoblast factors in RAW 264.7 cells. Consequently, these results indicate that EU polysaccharides exert an anti-inflammatory effect by inhibiting the expression of various proinflammatory cytokines. However, evidence of anti-inflammatory activity is still scarce, and in-depth *in vivo* studies and clinical trials are needed. The precise mechanism underlying the anti-inflammatory action requires further investigation. Current research on the anti-inflammatory effects of EU polysaccharides focuses on bark extracts, with no studies on leaf extracts. More research on leaf extracts is needed to support the future application of EU polysaccharides. We believe that EU polysaccharides may have potential as natural drug candidates in suppressing inflammatory responses in the future and make more contributions to human health.

### 5.4 Anti-complementary activity

The complement system is a complex biochemical reaction system and constitutes a crucial component of the innate immune system, which is the cornerstone of the body’s defence network (Samuelsen et al., 1999). Therefore, we need to pay more attention to the protection and repair of the complement system to safeguard our health. Recently, medicinal plants have been extensively studied for the extraction of polysaccharides, which exhibit remarkable efficacy and minimal toxicity. These polysaccharides have proven to be invaluable as raw materials for the development of novel natural complement inhibitors for the treatment of associated diseases caused by overactivation of complement, which has attracted increasing attention (Xia et al., 2019; Huo et al., 2020; Xia et al., 2020). Zhu et al. (2008), Zhu et al. (2009) extracted polysaccharides (EWDS-1 and EWDS-2) from the EU Cortex by the hot water method. The CH50 and AP50 values of EWDS-1 were 203 ± 20 μg/mL and 45 ± 8 μg/mL, respectively. The CH50 and AP50 values obtained for EWDS-2 were 282 ± 11 μg/mL and 144 ± 17 μg/mL, respectively. Preliminary mechanistic studies showed that EWDS-1 and EWDS-2 effectively suppressed complement activation through their interactions with C1q, C1r, C1s, C2, C3, C4, C5, and C9, thereby providing a crucial regulatory mechanism for both the classical and alternative pathways. Therefore, EWDS-1 and EWDS-2 have potential value in treating diseases linked to overactivation of the complement system. However, to date, there are few reports on the anti-complementary effect of EU polysaccharides, and the structure-activity relationship remains unclear, so it cannot be confirmed that only EWDS-1 and EWDS-2 have this activity. Experiments on EU polysaccharides’ anti-complementary activity is needed soon to show their potential in treating diseases from complement overactivation.

### 5.5 Hepatoprotective activity

Damage to the liver can result in cirrhosis, fibrosis, steatohepatitis, and even cancer. A plethora of studies have elucidated that polysaccharides derived from plants have low toxicity and outstanding effects on liver protection (Yuan et al., 2019; Qu et al., 2020). EU polysaccharides are no exception. Gao et al. (Gao et al., 2020) found that EU polysaccharides can effectively alleviate liver injury in the context of HIRI. Ischaemic liver tissue and serum were collected for the detection of biochemical indices and pathological damage to liver tissue. The data showed that the serum concentrations of aspartate aminotransferase, alanine aminotransferase, tumor necrosis factor-*α*, and IL-1*β* were significantly decreased after EU polysaccharide pretreatment, the liver malondialdehyde level was markedly decreased, while the enzymatic activity of superoxide dismutase was significantly increased, and a remarkable reduction in the liver necrotic area was observed. The specific mechanism involves alleviating hepatic injury by reducing reactive oxygen species (ROS) levels and inhibiting the activation of the TLR-4-NF-κB pathway. Clinically, serum markers such as alanine transaminase (ALT) and aspartate transaminase (AST) serve as valuable indices for the assessment of hepatoprotective potential (Feng et al., 2020). According to existing studies, EU polysaccharides significantly reduce ALT and AST levels, and EU polysaccharides exhibit a hepatoprotective effect on hepatic ischaemia‒reperfusion injury by improving antioxidant capacity and attenuating oxidative stress injury. Therefore, EU polysaccharides may be assumed to be an effective medicine for liver injury. However, as the results are based on only a few scholars and have not been tested in a broader clinical population, further studies are needed to confirm its safety and efficacy.

### 5.6 Other activities

Other biological activities have also been identified in EU polysaccharides. In the first instance. Xia and Piao (2010) found that polysaccharides from EU leaves can improve the muscle function of toads and have anti-fatigue properties. In comparison to that in the control group, the time needed for maximum muscle contraction in the polysaccharide group significantly decreased (*P*< 0.05), indicating that muscle fatigue was alleviated (*P*< 0.05; *P*< 0.01). Second. Jiang et al. (2011) found that EU polysaccharides demonstrated a favourable influence on CJ-S131-induced systemic lupus erythematosus syndrome in BALB/c mice. Treatment with EU polysaccharides (from *Eucommiae cortex*) reduced immunoglobulin deposition and albuminuria, protecting the kidneys from glomerular damage. Increases in serum autoantibodies and total IgG levels were also effectively suppressed. EU polysaccharides have the potential to be novel drugs for the treatment of autoimmune disorders. Moreover, Sun et al. (Sun et al., 2022) found that EU polysaccharides inhibited subsequent neuroinflammation by inhibiting the expansion of *Escherichia coli*, regulating the intestinal microbiome and tryptophan metabolism, and reducing the concentration of lipopolysaccharides (LPS) in colon contents and serum. In addition, oral EU polysaccharides inhibit peripheral Kyn pathways and rescue glutamate-induced neuroexcitatory toxicity. These findings suggest that EU polysaccharides have significant neuroprotective potential. Overall, previous research has demonstrated that EU polysaccharides have various biological activities, which was consistent with other natural polysaccharides. Exploring the structure-activity relationship of EU polysaccharides in greater depth will advance the development of potential health products and clinical drugs. Moreover, many the biological effects of EU polysaccharides need to be further studied to elucidate possible structure-activity.

## 6 Structure-bioactivity relationship

The chemical structures of EU polysaccharides are the basis of their pharmacological activities. Detailed correlation between structures and activities of EU polysaccharides obtained is of great importance for providing theoretical basis and future directions for exploring the treatment of diseases with EU polysaccharides. However, with these inadequate studies on polysaccharides derived from EU, the current understanding of the structure-activity relationship of EU polysaccharides is still lacking and somewhat inconsistent.

Currently, it is widely recognized that molecular weight and monosaccharide composition are two of the most important factors affecting the biological activities of polysaccharides (Mo et al., 2022). Liu et al. (2023b) believed that differences in antioxidant activity among EUP1, EUP2 and EUP3 purified from EU leaves might result from the differences among molecular weight, branching degree, monosaccharide composition and connection position of monosaccharide residues. When the molecular weights of these three components were within a certain range, the higher the molecular weight was, the lower the scavenging capacity of DPPH and OH radical would be. Obviously, EUP1 with the largest molecular weight (1.51 × 10^5^ Da) of the three had the weakest scavenging ability. Nevertheless, studies have found that the correlation between molecular weight and bioactivities of polysaccharides is not always straightforward (Chen H. et al., 2021). Zhu et al. (2008), Zhu et al. (2009) extracted and isolated from stem barks of EU, obtaining two homogeneous polysaccharides EWDS-1 and EWDS-2, with molecular weights of 2000 and 1000–2000 kDa, respectively. The CH50 (282 ± 11 μg/mL) and AP50 (144 ± 17 μg/mL) of EWDS-2 were significantly higher than EWDS-1 with the CH50 and AP50 values of 203 ± 20 μg/mL and 45 ± 8 μg/mL, suggesting that EWDS-2 with smaller molecular weight exhibited weaker anticomplement activity. In addition to molecular weight, it would be of interest to investigate regarding the relationships between monosaccharide composition and activity of polysaccharides, because different monosaccharide compositions of polysaccharides may contribute to different bioactivities. In the same study by Liu et al. (2023a) mentioned above, EUP1 exhibited the weakest free radical scavenging ability. It was found that the mole ratio of Rha 3.66 mol% in EUP1 was much lower than that in EUP2 (7.92 mol%) and EUP3 (18.67 mol%). In agreement with the literature, therefore, Liu et al. believed that Rha was the most important factor related to the free radical scavenging ability of polysaccharides.

In general, biological activities of EU polysaccharides can be also affected by their other structural features such as branching, glycosidic bond and chain conformation. Studies on a detailed structure–activity relationship in EU polysaccharides still needs further investigation. Thus, there is an urgent need to conduct substantial numbers of studies for comprehensive understanding on the structure−activity relationship in EU polysaccharides, which will facilitate the development of EU food supplements and medications.

## 7 Discussion

### 7.1 Applications

**FIGURE 4:**
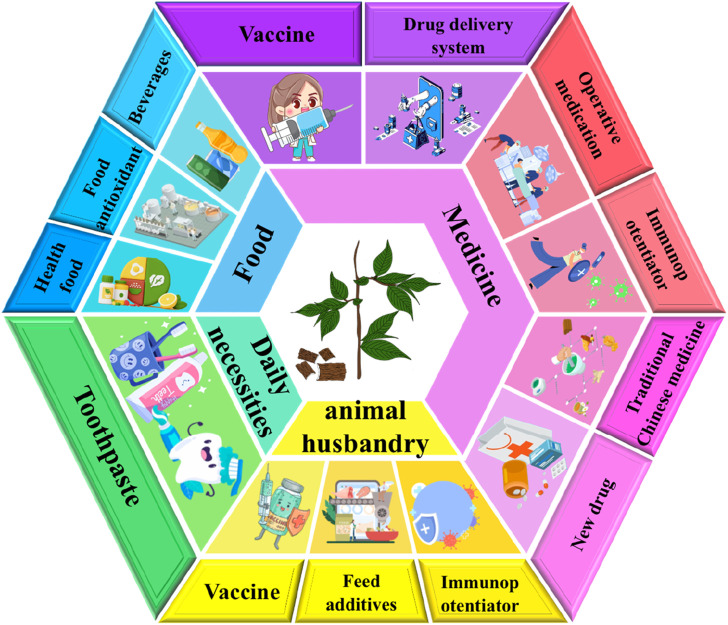
Current application and future development trend of *Eucommia ulmoides* polysaccharides.

**FIGURE 5:**
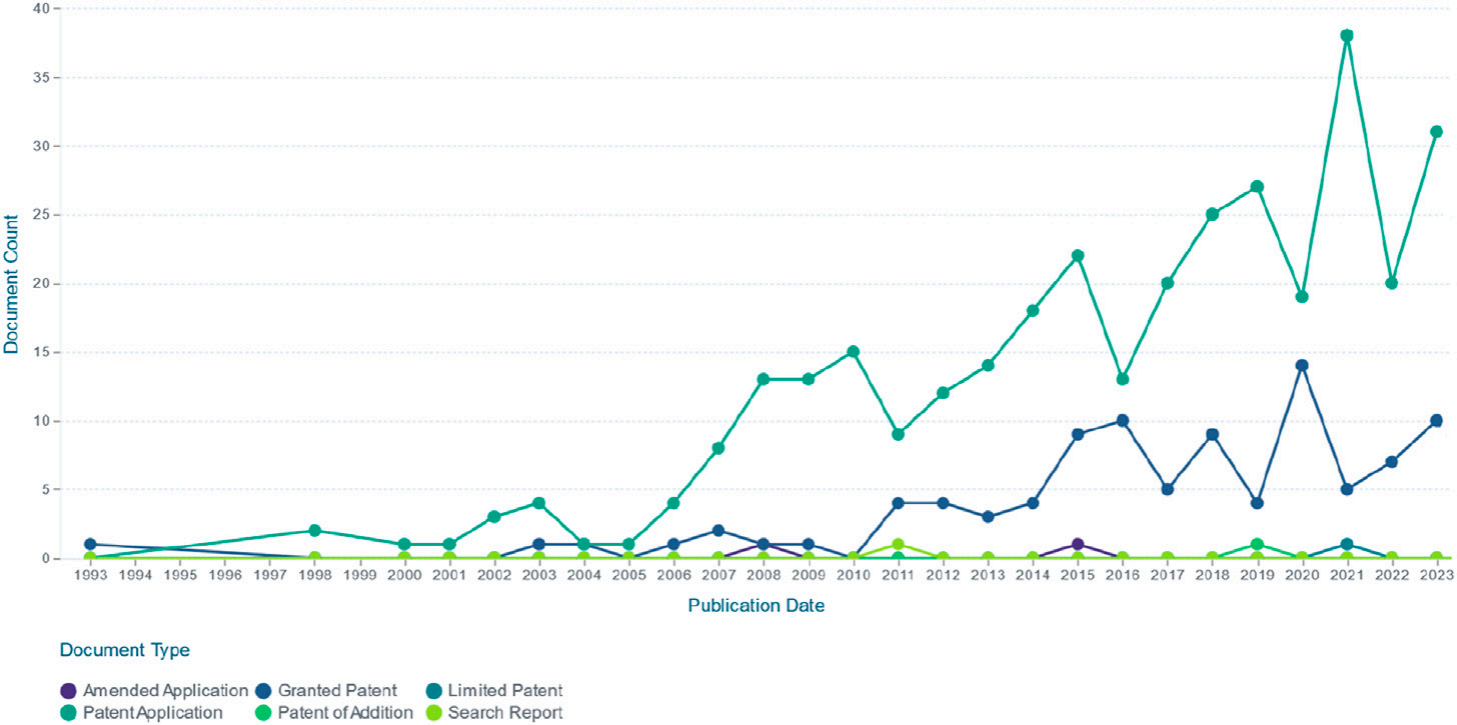
Final dataset of the number of *Eucommia ulmoides* polysaccharide-related patents per year globally up to 2023. (Source: http://www.lens.org).

EU polysaccharides are rich in biological activities and have broad application prospects. Currently, EU polysaccharides are not only recognized for their ability to prevent and treat diseases, but also widely used in animal husbandry (as veterinary pharmaceutical biologics, livestock immune adjuvants, feed additives, etc.) and food industry (as dietary supplements, nutrition enhancers, etc.). The relevant patent information is shown in Table 4. The application status and future development of EU polysaccharides are shown in Figure 4 (made with assets from Freepik.com). Meanwhile, Figure 5 details the current status of EU related invention patents, including the annual global application and publication of patents for EU and its polysaccharides. In addition, we also inquired about the application for the approval of EU products in the State Administration for Market Regulation, as detailed in Table 5.

**TABLE 4:** Application of *Eucommia ulmoides* polysaccharides related patents.

NO	Application	Name of the invention	Main composition	Effect	Publish number (A)
1	Daily necessities	The invention relates to a toothpaste with the effect of whitening, removing stains and improving tooth sensitivity and a preparation method	Polysaccharides of *E. ulmoides*	Restore enamel health	CN113599285
2	Biomedicine	*E. ulmoides* polysaccharide strontium complex and its preparation method and application	Polysaccharides of *E. ulmoides*	Promote cell proliferation	CN110604742
3	Health product	A composition that modulates human microbiota and immune function	Polysaccharides of *E. ulmoides*	Regulates human microbiota and immune function	CN102813113
4	Silver nanoparticles	Synthesis method of *E. ulmoides* polysaccharides silver nanoparticles, synthesized *E. ulmoides* polysaccharides silver nanoparticles and their applications	Polysaccharides of *E. ulmoides* bark and leaves	Hemostasis, antibacterial and anti-inflammatory	CN113527769
5	Nanomaterials	Method of preparing selenium nanoparticles using *E. ulmoides* polysaccharide and the prepared selenium nanoparticles	Polysaccharides of *E. ulmoides* bark or leaves	Stabilize and enhance the biological activity of selenium nanoparticles	CN113456831
6	Veterinary medicine	A soluble granule for improving pig immunity and a preparation method	Polysaccharides of *E. ulmoides* leaves	Immune regulation	CN110812389
7	Feed additive	An additive that increases disease resistance to promote growth in pigs	Polysaccharides of *E. ulmoides* leaves	Promotes the proliferation of beneficial microorganisms in the animal gut, Immune regulation	CN105707437
8	Functional food	*E. ulmoides* leaves polysaccharides with antitumor activity, extraction and isolation methods, and their application in the preparation of supplements for antitumor drugs	Polysaccharides of *E. ulmoides* leaves	Anti-tumor	CN114989323
9	Traditional Chinese medicine	The invention relates to a preparation method of *Physalis calyxseuf* ructus extract for treating wound healing and scald	Polysaccharides of *E. ulmoides* leaves	Immune regulation, antioxidant	CN114159508
10	Biological products for veterinary use	African swine fever virus vaccine strains and vaccines containing vaccine strains	Polysaccharides of *E. ulmoides* bark	Anti-virus	CN110393798
11	Food	The invention relates to a *Elaeagnus sarmentosa* compound fruit and vegetable juice and a preparation method	Polysaccharides of *E. ulmoides* bark	Immune regulation	CN108967760
12	Clinical implant surgery	An implant containing a polysaccharide coating that can bind growth factors and a preparation method	Polysaccharides of *E. ulmoides* bark	Binding growth factors	CN108478298
13	Traditional Chinese medicine	A *E. ulmoides* targeting agent for colon cancer and a preparation method	Polysaccharides of *E. ulmoides* bark	Anti-tumor	CN104434950
14	Immune adjuvants for livestock and poultry	Chinese medicine Morus alba and *E. ulmoides* polysaccharides immunopotentiator and its application	Polysaccharides of *E. ulmoides* bark	Immune regulation	CN103768594

**TABLE 5:** Applications in health foods related to *Eucommia ulmoides*.

Approval number	Name	Category	Function
G20050865	Meidakang Brand Sheep Kidney Hardy Rubbertree Chinese Boxthorn Oral Liquid	Health Food	Relieve Physical Fatigue
G20110543	Tianyuankang Brand American Ginseng Mongolian Milkvetch Hardy Rubbertree Capsule	Health Food	Relieve Physical Fatigue
G20140918	Renyibao Brand Ginseng Hardy Rubbertree Solomon’s Seal Oral Liquid	Health Food	Relieve Physical Fatigue
G20200030	Jimei Brand Bishop’s Hat Hardy Rubbertree Tablet	Health Food	Relieve Physical Fatigue
G20200462	Jishengyuan Brand Hardy Rubbertree Jiaogulan European Dogbane Capsule	Health Food	Boost Immunity
G20100508	Jinqi Brand Roseroot Stonecrop Chinese Boxthorn Hardy Rubbertree Tea	Health Food	Boost Immunity
G20100185	Guojianglong Brand Cherokee Rose Hardy Rubbertree Vinum	Health Food	Boost Immunity
G20120373	Jijia Brand Hardy Rubbertree Sea Buckthorn Capsule	Health Food	Boost Immunity, Relieve Physical Fatigue
G20130563	Wudangzixiao Brand Hardy Rubbertree Solomon’s Seal Vinum	Health Food	Boost Immunity, Relieve Physical Fatigue
G20110321	Huiyuannonggu Brand Hardy Rubbertree Beverages	Health Food	Boost Immunity, Relieve Physical Fatigue
G20100749	Baibang Brand European Dogbane Hardy Rubbertree Kudzu Danshen Safflower Flos Sophorae Immaturus Granules	Health Food	Reduce Blood Press
G20100436	Jianerma Brand Kudzu Hardy Rubbertree European Dogbane Tea	Health Food	Reduce Blood Press
G20041320	Zhengbenjingfang Brand Hardy Rubbertree Kudzu Capsule	Health Food	Reduce Blood Press
G20100054	Meiluo Brand Gastrodia Semen Ziziphi Spinosae Water Plantain Hardy Rubbertree Capsule	Health Food	Reduce Blood Press, Improve Sleep
G20120245	Fuzhen Hardy Rubbertree Squirrel’s Foot Fern Bishop’s Hat Capsule	Health Food	Increases Bone Mineral Density
G20140620	Maojitang Brand Squirrel’s Foot Fern Hardy Rubbertree Extractive Capsule	Health Food	Increases Bone Mineral Density
G20120435	Jiuzhang Brand Hardy Rubbertree Honeysuckle Reishi Shell-broken Spore Powder Chewable Tablets	Health Food	Resistance to Chemical Liver Injury
G20120100	Linlanhua Brand Dendrobium Kudzu Hardy Rubbertree Capsule	Health Food	Resistance to Chemical Liver Injury, Boost Immunity
(1997) No. 072	Lijian Brand Hardy Rubbertree Beverages	Health Food	Anti-fatigue
G20040051	Heshou Brand Hardy Rubbertree Tea	Health Food	Anti-fatigue
G20141164	Wansongtang Brand Ginkgo Hardy Rubbertree San Qi Tea	Health Food	Reduce Blood Lipid
G20050086	Hongheyuan Brand Hardy Rubbertree Capsule	Health Food	Boost Immunity, Improve Sleep

#### 7.1.1 Field of medicine

With the deepening of research on plant polysaccharides, a large number of plant polysaccharides have been developed and applied in pharmaceutical industry, and become a hot spot in the study of natural active ingredients (Elango et al., 2023). EU polysaccharides are no exception, in the pharmaceutical industry, EU polysaccharides are utilized in traditional Chinese medicine preparations due to their biological activities. Studies have found that strontium plays an important role in bone formation, and it can promote the proliferation of osteoblasts and pluripotent stem cells and improve bone metabolism (Liu et al., 2023). However, free strontium ions are toxic *in vivo*, and the application of strontium is difficult to develop. The strontium complex of EU polysaccharides can avoid the toxicity of free strontium ions. At the same time, the prepared EU polysaccharides strontium complex also enables the effective release of strontium in the body, plays the role of strontium in reducing inflammation and osteocyte proliferation, and can be a good auxiliary treatment of osteoarthritis. In addition, the above polysaccharides strontium complex can change the immune environment of bone, promote the proliferation and differentiation of bone repair cells, and play an important role in bone repair. In addition, EU polysaccharides can also be used as anti-tumor drugs and active ingredients. There are *in vivo* anti-tumor tests that show, *Eucommiae folium* polysaccharide can effectively inhibit the growth of solid tumor in mice, protect immune organs and liver tissues of mice, induce tumor cell apoptosis, block tumor cells in S phase, prevent their further proliferation, destroy mitochondrial membrane potential of cells, and induce tumor cell apoptosis through mitochondrial pathway in a certain dose dependent manner. Therefore, polysaccharides from *Eucommiae folium* can be used in the preparation of supplements for antitumor drugs. It is worth mentioning that EU polysaccharides can also be used to prepare a targeted preparation for the treatment of colon cancer. This EU targeted preparation contains EU polysaccharides, which can evenly distribute the drug release throughout the colon, increase the drug concentration in the diseased part of the colon, reduce the dose, reduce the stimulation of the gastrointestinal tract and the toxic side effects caused by the drug absorption, so as to exert the best curative effect and facilitate the treatment. Because polysaccharides from EU can be used as a sugar source of colon flora and can be specifically degraded, it is an ideal choice for the treatment of colon cancer. Furthermore, polysaccharides from *Eucommiae folium* have been used as a paste for wound healing and scalding due to their immunomodulatory activity. Polysaccharides extract from *Eucommiae folium* can enhance specific immunity, activate T and B lymphocytes, promote the proliferation of beneficial bacteria, and have the ability to remove hydroxyl radical and superoxide anion radical, promote cell growth, and have good therapeutic effect on wound healing and scalding.

#### 7.1.2 Field of functional food

With the progress of science and technology and the development of society, environmental pollution has intensified, air pollution, water pollution and food safety threaten every family, and at the same time, modern people have a fast pace of life, great work pressure and irregular diet, resulting in more and more sub-health people, so functional food has been favored by more and more people (Yu et al., 2024). At present, some health food containing EU polysaccharides has been developed. For example, a compound fruit and vegetable juice not only have good taste, clear color, long-term storage without discoloration, but also is rich in nutrition, can enhance human immunity, prevent various diseases, and help solve human sub-health problems. It contains EU polysaccharides can enhance the body’s immunity, and play a role in lowering blood sugar, anti-oxidation and anti-liver fibrosis. At the same time, it can improve the taste of fruit and vegetable juice and make it easier for consumers to accept. In addition, EU is added to oral liquids, beverages, or alcohol. Because the addition of EU makes these products have the effect of relieving body fatigue and enhancing immunity. It is worth mentioning that *Eucommiae folium* tea has also been developed as a healthcare product, which can enhance immunity, anti-fatigue, and assist in lowering blood pressure and blood lipids. Of course, in addition to drinking products, EU extract is also added to some chewable tablets and capsules to develop health products with the effects of strengthening immunity, anti-fatigue, anti-chemical liver damage, increasing bone density, improving sleep, etc. It can be seen that EU and its polysaccharides have great potential in the development of functional food and can bring good commercial value.

#### 7.1.3 Other fields

With the deepening of EU research, EU polysaccharides have also been used in animal husbandry. First, EU polysaccharides can be used as vaccine adjuvants. The patented research shows that the vaccine containing vaccine strain prepared with the venom and polysaccharides of EU can prevent and control African swine fever, and has high popularization and application value. Another kind of animal immune adjuvant containing EU polysaccharides can significantly stimulate the proliferation of chicken lymphocytes, increase the titer of serum antibodies, and enhance cellular and humoral immunity of chicks *in vitro*. It can be seen that EU polysaccharides have broad application prospects in the field of vaccine adjuvant. Secondly, EU polysaccharides can also be used as feed additives to promote the growth of pigs and improve the disease resistance of piglets. Or it can be added into veterinary drug granules to comprehensively adjust and play the immune function and adaptability of pig’s own body, so as to significantly improve the disease resistance of pig, improve the health status of pig, play the role of disease prevention and treatment, reduce the morbidity and mortality of pig, and help improve the breeding efficiency and breeding quality. In addition, EU polysaccharides also have relevant applications in the field of daily necessities. A kind of toothpaste has the effect of whitening and removing stains and improving the sensitivity of teeth. The polysaccharide of EU is added to the toothpaste, which promotes the diphosphoglyceric acid to further play its role, plays a promoting role in restoring the health of tooth enamel and plays a whitening effect. Therefore, we believe that in the future, EU polysaccharides can be added to more daily necessities, such as creams, masks, etc., to enhance the whitening effect of products.

### 7.2 Resource utilization

EU is a unique tree species in China. This species has been employed as a medicine for the treatment of various diseases for a long time, and its medicinally functional plant part is mainly bark. However, harvesting the bark for medicinal use requires long growing periods, the tree itself is easily damaged during the harvesting process, and processing is relatively complex compared to that of the leaves, resulting in a waste of resources to a certain extent. There have been studies on replacing EU bark with leaves in some applications. According to the literature, the current research on EU polysaccharides is mainly focused on the bark and leaves of EU. In this research work, we explored whether polysaccharides from EU leaves could be used as a direct substitute for polysaccharides from EU bark. The results suggest that both EU bark polysaccharides and EU leaf polysaccharides have immunomodulatory, antioxidant and anti-inflammatory effects. In terms of immunomodulatory activity, EU leaf polysaccharides could play an immunomodulatory role by inducing DC maturation, promoting lymphocyte proliferation, and stimulating macrophages to produce NO, TNF-α, IL-6 and other immunomodulatory mediators, while EU bark polysaccharides could play an immunomodulatory role by stimulating B lymphocytes and DC cells. Further comparison of antioxidant activity showed that EU leaf polysaccharides exhibited a notable scavenging effect on DPPH, ABTS and hydroxyl radicals, while EU bark polysaccharides only had a notable scavenging effect on DPPH radicals. In addition, EU leaf polysaccharides have anti-fatigue and hepatoprotective pharmacological activities, and EU bark polysaccharides have anti-complementary and neuroprotective effects. Based on current applications, EU leaf polysaccharides and EU bark polysaccharides have been used in the pharmaceutical and food industries due to their immunomodulatory activities, and EU leaf polysaccharides have also been used in pharmaceuticals due to their antioxidant effects. All things considered, EU leaf polysaccharides may replace EU bark polysaccharides in immune regulation and antioxidant applications and be used in the pharmaceutical and food industries. These researches are important for promoting the conservation and sustainable use of EU plant resources. It is expected that soon, EU leaf polysaccharides may be a suitable substitute for EU bark polysaccharides and will be widely used in nutritional foods and natural medicine to promote human health.

### 7.3 Limitations of the current research

Despite the research of EU polysaccharides has come a long way, currently many continuing challenges and limitations for researchers still remain ahead to facilitate practical application.

Firstly, although studies have shown that EU polysaccharides exhibited diverse bioactivities, their underlying molecular mechanisms with inherent complexity remain elusive. At present, it is sobering to note that studies on these bioactivities of EU polysaccharides are still in the early stage. What is more, few data currently exist on the safety and efficacy of EU polysaccharides in clinical trials. Therefore, further studies are needed for application of molecular approaches to further explore the underlying mechanisms by using “omics”, such as genomics, proteomics and metabolomics.

Secondly, more detailed advanced structural features of EU polysaccharides were not very well known nor well studied. Further adequate investigation of EU polysaccharides should be considered on glycosidic linkage types, glycosidic linkage sequence, glycosidic linkage configuration, branch point position and conformation, which can be fully characterized by various classical approaches, such as FT-IR, GC–MS, NMR, X-ray diffraction, AFM, and methylation analysis.

Thirdly, deep insights into structural and functional properties of EU polysaccharides remain lacking, which could hinder further development and novel biological and pharmacological applications. Different medicinal parts may have different functions, thus to easily observe more commonalities and differences among EU polysaccharides researchers could do more work on comparing the structure features and activities of polysaccharides derived from more other EU medicinal parts, such as roots, fruits and flower. These results will be helpful to further elucidate the relationship between the structure and function of EU polysaccharides in the future.

Fourthly, in spite of good performance by EU polysaccharides in biological activities and broad application prospects, there are relatively scarce studies of EU polysaccharides in cosmetics. More efforts should be exerted toward great potential application not only in the areas of biomedicine and food industries but also in the areas of cosmetics. Therefore, there is an urgent need to develop a green sustainable standardized extraction and purification techniques that are economical, efficient and environment-friendly for high-quality EU polysaccharide products. So far, however, preparation of EU polysaccharides is restricted to laboratories only. As a result, preparation process for EU polysaccharides needs to be further optimized to offer scalable and reproducible manufacturing processes for commercial use.

In summary, EU has been widely used in food, health products and cosmetics, and EU polysaccharides have great potential for a wide range of industrial, biomedical and clinical applications with broad application prospects. Although there is still a long way to go to reach this goal, it is believed that in the coming years the product containing EU polysaccharides will assume a place in the health product market.

## 8 Conclusion and future prospectives

In this review, recent research progress in extraction, purification, structure, various pharmacological activities, structure-activity relationship and applications of polysaccharides derived from two different medicinal parts (bark and leaves) of EU was systematically summarized. Additionally, resource utilization and the limitations of the current research were also discussed. EU is a precious medicinal plant widely distributed in China. Its bark and leaves can both be used as medicinal remedies nourishing liver and kidney and strengthening muscles and bones since ancient times. To date, multiple extraction and purification methods have been used obtaining a total of about 12 and nine polysaccharides from EU bark and leaves, respectively. These EU polysaccharides have various important biological activities such as neuroprotective, antioxidant, immunomodulatory, anti-inflammatory, anti-complementary, anti-fatigue and anti-liver injury bioactivities and a beneficial effect on systemic lupus erythematosus. Such bioactivities prompted EU polysaccharides to have further diverse applications in medicine, functional foods, and animal husbandry, showing significant commercial potential and broad application prospects currently and in the future. These researches also suggest that EU leaf polysaccharides with broader immunomodulatory, antioxidant, and pharmacological effects could potentially replace EU bark polysaccharides in pharmaceutical and food industries. Such substitution would potentially promote the sustainable use of EU plant resources and enhance human health through their application in nutritional foods and natural medicine.

## Abbreviations

AE, Alkali Extraction; Ara, Arabinose; EU, Eucommia ulmoides; Fru, Fructose; Fuc, Fucose; Gal, Galactose; GC, Gas Chromatography; Glc, Glucose; HPLC, High Performance Liquid Chromatography; MAE, Microwave-Assisted Extraction; Man, Mannose; Mw, Molecular Weight; NMR, Nuclear Magnetic Resonance Spectroscopy; Rha, Rhamnose; UAE, Ultrasound-Assisted Extraction; UV, Ultraviolet Visible Spectroscopy.
